# Judicialization of access to medicines in four Latin American countries: a comparative qualitative analysis

**DOI:** 10.1186/s12939-019-0960-z

**Published:** 2019-06-03

**Authors:** Claudia Marcela Vargas-Pelaez, Marina Raijche Mattozo Rover, Luciano Soares, Carine Raquel Blatt, Aukje K. Mantel-Teeuwisse, Francisco Augusto Rossi, Luis Guillermo Restrepo, María Cristina Latorre, José Julián López, María Teresa Bürgin, Consuelo Silva, Silvana Nair Leite, Mareni Rocha Farias

**Affiliations:** 1 Programa de Pós-Graduação em Farmácia, Universidade Federal de Santa Catarina, Campus Universitário Trindade, Florianópolis, Santa Catarina Brazil; 2 World Health Organization Collaborating Centre for Pharmaceutical Policy & Regulation, Utrecht Institute for Pharmaceutical Sciences (UIPS), Universiteitsweg 99, Utrecht, The Netherlands; 3 Departamento de Ciências Farmacêuticas. Centro de Ciências da Saúde, Universidade Federal de Santa Catarina, Campus Universitário Trindade, Florianópolis, Santa Catarina Brazil; 4 Programa de Pós-Graduação em Saúde e Meio Ambiente, Universidade da Região de Joinville, Rua Paulo Malschitzki, 10 - Bom Retiro, Joinville, Santa Catarina Brazil; 5 Departamento de Farmacociências, Universidade Federal de Ciências da Saúde de Porto Alegre, Rua Sarmento Leite, 245 - Cidade Baixa, Porto Alegre, Rio Grande do Sul Brazil; 6 Fundación IFARMA, Carrera 13 # 32-51, Torre 3, Oficina 1115, Bogotá, Colombia; 7 Departamento de Farmacia, Universidad Nacional de Colombia, Carrera 30 No. 45-03, Edificio 450, Oficina 214, Bogota D.C, Colombia; 8 Corporación de Investigación MEGA 2, Ricardo Matte Perez 450, Santiago, Chile; 9 Independent researcher, Ciudad Autónoma de Buenos Aires, Argentina

**Keywords:** Right to health, Essential medicines, Access to medicines, Lawsuits, Argentina, Brazil, Chile, Colombia

## Abstract

**Background:**

The valuation of medicines as health needs vary depending on the stakeholders involved (users, prescribers, managers, etc.) and their expectations. These factors modulate the role of medicines as a health need and influence access to medicines, and could be useful to explain the rising of Judicialization of access to medicines.

**Aim:**

To conduct a comparative analysis of the causes and consequences of judicialization of access to medicines in Argentina, Brazil, Colombia and Chile from the perspective of medicines as health needs.

**Methods:**

A qualitative, cross-country study was carried out in these 4 countries. Semi-structured interviews were conducted with 50 representatives of the different stakeholders involved in the judicialization of access to medicines, including Executive branch, Judiciary, health system managers, patient organizations. The interviews were audio-recorded and transcribed verbatim. Thematic analysis used a framework approach based on the theoretical model for medicines as health needs.

**Findings:**

Representatives from Argentina, Brazil and Colombia considered judicialization of access to medicines as a widespread phenomenon in their respective countries. Meanwhile in Chile, the respondents highlighted that most lawsuits related to the right to health were filed against private insurers because of unjustified increases in the insurance premiums. The comparative analysis showed that judicialization of access to medicines emerged in the four countries regardless of the constitutional protection or the health system population coverage. Among the causes were mentioned difficulties in guaranteeing access to covered medicines and the influence of pharmaceutical marketing on needs assessment and prescription behaviours. The interviewees highlighted the pressure to health system managers to fulfil their responsibilities as a positive impact of litigation. In contrast, the funding of medicines without evidence of efficacy or safety was considered a negative impact. Only in Brazil, judicialization has had impact on R&D policies. In Colombia, litigation also encouraged the recognition of the right to health as a fundamental right and the development of policies for controlling medicines prices.

**Conclusion:**

The results suggest that applying the adopted theoretical model creates the possibility of identifying critical points to guide policy makers to improve the health systems performances and to control lawsuits for access to medicines.

**Electronic supplementary material:**

The online version of this article (10.1186/s12939-019-0960-z) contains supplementary material, which is available to authorized users.

## Background

Medicines are perceived as a health need once they are considered valuable goods. This valuation can vary depending on the stakeholders involved (users, prescribers, managers, etc.) and their expectations, economic and political interests [1]. Valuation differences materialize in the process of incorporation of certain technologies over others in the health system’s coverage, and lawsuits for access to medicines uncovered by the health systems. In this way, the judiciary as a guarantor of the right to health obtained an active role in the recognition of medicines as health needs and became a modulator of public policies for access to medicines. This phenomenon is known as Judicialization of access to medicines [2].

Judicialization is defined as a complex phenomenon that involves technical-scientific, legal and social aspects. In the literature two approaches to this phenomenon can be found: normative and social. Within the normative approach Judicialization is understood as the interference of the Judicial Power in the Executive Power, whereas within the social approach Judicialization is considered a form of citizen participation [3].

Judicialization of access to medicines has risen in the Latin American region, mainly in Colombia and Brazil [4, 5], and the magnitude of the phenomenon is growing in countries such as Argentina and Chile [6], despite the differences in the socio economic indicators, (Table 1), the legal recognition of the right to health (Table 2) and health system organization among these countries [2]. A brief description on how the health systems in these four countries functioned at the moment this study was carried out can be found in the Additional file 1.

**Table 1:** General information about the studied countries

Country	Argentina	Brazil	Chile	Colombia
Population (2014)^(a)^	42,981,515	204,213,133	17,613,798	47,791,911
Administrative division	23 provinces 1 Autonomous City	26 states, 1 federal district	15 regions	32 departments 1 capital district
Life expectancy at birth (2014)^(a)^	76	75	79	74
Under-5 mortality per 1000 live births (2014)^(a)^	12	16.2	8.1	16.2
GDP per capita (Current USD) (2014)^(a)^	12,245	12,026	14,794	7913
GDP per capita PPA (2014)^(a)^	19,400	15,880	22,210	12,950
Gini index (2014)^(a)^	41.4	51.5	47.3	52.8
HDI Rank (2014)^(b)^	0.808	0.757	0.830	0.720
THE as % of GDP (2014)^(c)^	8	11	8	6
Government expenditure on health as % of THE (2014)^(c)^	77	34	59	64
Out-of-pocket on health as % of THE (2014)^(c)^	15	45	34	20
Government expenditure on health per capita PPP (2014)^(c)^	1255	593	1057	498

*Abbreviations: GDP* Gross Domestic Product, *HDI* Human Development Index, *THE* Total Health Expenditure, *PPP* purchasing power parity value. Sources: (a) World Bank indicator. Retrieved from: data.worldbank.org [Accessed: 10 Mar 2019]. (b) United Nations Development Programme – Human Development Reports. Retrieved from: http://hdr.undp.org/en/countries [Accessed: 10 Mar 2019]. (c) WHO, Global Health Expenditure Database. Retrieved from: http://apps.who.int/nha/database/Select/Indicators/en. [Accessed: 10 Mar 2019]

**Table 2:** Right to health and pathways to resort the Judiciary for protecting it in Argentina, Brazil, Chile and Colombia

Right to health	Access to medicines	Pathways to resort the Judiciary
Argentina		
*National Constitution, Article 42* “Consumers and users of goods and services have the right to the protection of their health, safety, and economic interests”. Each province defines in its Constitution the recognition of the right to health in its territory.	*Decree 492/95, Article 1:* “The beneficiaries of the agents of the National Health Insurance System, covered by Article 1 of Law No. 23.660, are entitled to receive medical care benefits established in the medical care program to be approved by the Ministry of Health and Social Welfare through the secretary of health policy and health regulations. This program will call the Compulsory Medical Program (PMO) and will be mandatory for all agents set forth above”. *Law 24.754, Article 1.* “From within 90 days of enactment of this law, companies or entities that provide prepaid medical services should cover at least in medical care plans the same” Mandatory Medical Plan (PMO) arranged to the *Obras Sociales*, as established by Law 23.660, 23.661 and 24.455, and their respective regulations. For the public sector, each province defines its own regulations on the coverage of medicines.	*Amparo.* It can be brought only to federal, civil and commercial tribunals. *Amparo* requires the intervention of a lawyer
Brazil		
*National Constitution, Article 196*: “Health is a right of all and a duty of the State and shall be guaranteed by means of social and economic policies aimed at reducing the risk of illness and other hazards and at the universal and equal access to actions and services for its promotion, protection and recovery.”	*Law 8080/1990, Article 6:* “… the Unified Health System - SUS also includes in its field of action: I - the execution of actions: (d) of integrated care, including pharmaceutical assistance”. *Law 12,401/2011, Article 1°* The integral therapeutic assistance referred to in point d of subsection I of art. 6 consists of: I - dispensing of medicines and products of health interest whose prescription is in accordance with the therapeutic guidelines defined in clinical protocol for the disease or health problem to be treated or, in the absence of the protocol, in accordance with the provisions of Art. 19-P;	Civil lawsuit It can be filed in any tribunal. Require the intervention of a lawyer. It could be individual or collective
Chile		
*National Constitution, Article 19 No. 9:* “The right to health protection. The State protects free and equal access to the actions for the promotion, protection and recovery of health and rehabilitation of the individual. It will also be responsible for coordination and control of health-related actions. It is the prime duty of the state is to ensure the implementation of health actions, whether undertaken by public or private institutions, in the form and manner prescribed by law, which may establish compulsory contributions. Every person shall have the right to choose the health care system that wishes to join, be it state or private.”	*Law N° 19.966, Article 2:* The General System of Guarantees shall also contain Explicit Health Guarantees concerning access, quality, financial protection and timeliness of the benefits provision associated with a prioritized set of programs, diseases or health conditions indicated by the corresponding decree. The National Health Fund (FONASA) and the Health Insurance Institutions (ISAPREs) shall mandatorily ensure such guarantees to their respective beneficiaries.	Protection resource. It only can be brought to the Supreme Court. Require the intervention of a lawyer.
Colombia		
*National Constitution, Article 49:* “Health care and environmental protection are public services charged to the state. To everyone is guaranteed access to health promotion, protection and recovery. The State organizes, manages and regulates the provision of health services and environmental sanitation to residents according to the principles of efficiency, universality and solidarity.”	*Law 100/1993, Article 156*. “Basic features of the general social security health. The general social security health shall have the following characteristics: *(d)* All members of the general system of social security health plan will receive a comprehensive health protection, with preventive care, medical-surgical and essential drugs, which will be called mandatory health plan.”	*Tutela* action It can be brought to any tribunal. Does not require the intervention of a lawyer

In short, between 2000 and 2014 Argentina, Brazil, Colombia and Chile took measures aiming to extend the coverage of their health systems and access to medicines. However, the population coverage is fragmented and depends on the socioeconomic conditions of the individual, mainly related to their labour status, health status and ability to pay. Argentina has the highest level of fragmentation, with seven different pathways to access the health system, followed by Chile where the population is stratified according to the income in the public sector and according to their ability to pay and their health status in the private sector. In Brazil, although all citizens have access to public health services in the Unified Health System (SUS by the name in Portuguese), the presence of the private health insurance creates fragmentation as regards ability to pay and health status. In Colombia, access to the health system depends mainly on the income level and labour status within the General Social Security in Health System (SGSSS by the name in Spanish), and the income level and health status in the case of private health insurance [2].

The fragmentation in the population coverage results in the fragmentation of the health services and technology coverage, since the medicines included in the benefit plan which people have the right to depends on their relationship with the health system. During the mentioned period, Argentina, Colombia and Chile implemented reforms aimed to reduce the differences in quality and access to health care and medicines between different health systems’ subsectors. In Argentina, the country that has more diversity in the list of covered medicines among and within the health system’s subsectors, the Mandatory Medical Plan (PMO by its name in Spanish) statement was an attempt to achieve equalization of the benefits for all beneficiaries in the social insurance sector; nevertheless since this is a minimum coverage, and the financial capacity of the *Obra Social* (OS) depends on the level of income of their affiliates, the inequality in the access to medicines remains [7]. In Colombia, the benefit plans of the contributory and subsidised regimes were equalized and updated; and in Chile the Explicit Guarantees in Health for the public sector in the National Fund of Health (FONASA by the name in Spanish) and the private insurance companies (ISAPREs by the name in Spanish) were established. In the case of Brazil, a reorganization of the Specialized Component of the Pharmaceutical Assistance of the SUS (CEAF by its name in Portuguese) took place, in which the funding and the list of the medicines coverage was updated [2].

Another important aspect that varies among the countries is the scope of the Health Technologies Assessment (HTA) agencies. While the Brazilian agency (CONITEC) and the Colombian agency (IETS) assess and advise about inclusion of medicines to be supplied for the entire or almost the entire population in their countries, the Argentinian agency (SUR) has a restricted scope because its decisions only bind to the national OS. In Chile was not created a specific agency for HTA.

Regarding the medicines funding, in the Argentinian context, as aforementioned, the SUR only covers national OS affiliates and covers only a list of medicines defined by the Health Superintendence, and in the public sector there are specific funds for high cost medicines, but they are fragmented and cover only some specific medications. In Chile, besides the fact that the Additional Coverage for Catastrophic Diseases (CAEC by its name in Spanish) does not have a specific coverage (but explicit exclusions), this fund covers only the open ISAPREs affiliates; and in the public sector there are other specific funds for high-cost medicines and health services which have limited economic capacity, and then, limited population coverage. It is expected that some of these problems might be resolved with the implementation of Law Ricarte Soto, which constitutes the first intent for reducing the financing fragmentation of the Chilean health system.

Finally, during the period 2000–2014, the focus of the public policies for access to medicines across the 4 studied countries changed from essential medicines to high-cost medicines, bringing potential regressive effects on the guarantee of the right to health especially for the most vulnerable people [2].

Access to medicines depends on complex and dynamic relationships among the health system’s stakeholders, and the health system’s organization and litigation for access to medicines are determined by social and political features [3]. Recently, a theoretical model was proposed to analyse how technical, political and social factors modulate the role of medicines as a health need and influence accessibility and access to medicines [1].

This model offers the opportunity to analyse the possible causes and consequences of litigation for access to medicines in these countries, from the stakeholders’ perspective, in order to better understand the phenomenon and collect information to work towards possible solutions to promote equitable access to medicines. Within this framework, this study aims to conduct a comparative analysis of the causes and consequences of judicialization of access to medicines in Argentina, Brazil, Colombia and Chile from the perspective of medicines as health needs.

## Methodology

A cross-country study including Argentina, Brazil, Chile and Colombia was carried out. In-depth semi-structured interviews were conducted in order to explore the perceptions of stakeholders regarding the possible causes and consequences of judicialization of access to medicines in the studied countries. Semi-structured interviews were conducted with 50 key actors linked to the different stakeholders involved in the judicialization of access to medicines phenomenon (Table 3). A key actor was defined here as an individual involved in the judicialization of access to medicines, working for the stakeholder during at least 1 year, who was willing to offer his or her expertise, opinions and knowledge to the object of study. The interviews were conducted in Argentina (Buenos Aires, La Plata), Brazil (Rio de Janeiro, Porto Alegre, Brasilia, and Sao Paulo), Chile (Santiago) and Colombia (Bogota), between August and December 2014.

**Table 3:** Number of interviewed respondents

Stakeholder	Argentina	Brazil	Chile	Colombia	Total
Executive^(a)^	2	1	0	5	8
Judiciary	3	3	0	2	8
Health system manager ^(b)^ (Manager)	6	3	2	2	13
Patient organization (Patient)	1	1	1	2	5
Health professional organization (Professional)	1	1	2	2	6
Other	5^(c)^	0	2^(d)^	3^(e)^	10
Total	18	9	7	16	50

Source: The authors

^(a)^ Executive: Ministry of Health, medicines regulatory agency, superintendence of health or healthcare services;

^(b)^ Health system managers: State Health departments (Brazil), *Obras Sociales* (OSs) (Argetnina), *Instituciones de Salud Previsional* (ISAPREs) (Chile), *Empresas Promotoras de Salud* (EPSs) (Colombia)

^(c)^ NGOs, Senator’s advisor, Expert in pharmaceutical marketing, Expert in public policies of health

^(d)^ Lawyer involved in judicial cases for access to medicines, University lecturer expert in health economics

^(e)^ NGOs, University lecturer expert in litigation for health right

The researchers from each country, who were involved in the study, helped to identify key actors as potential participants. Additional respondents were identified through the snowball technique [8]. Respondents were invited to participate in the study by means of e-mail, telephone or personally. All the interviews were conducted as individual face-to-face interviews and, in almost all the cases, they happened at the participants’ workplace.

The interview script included two general questions: *(a)* “In your opinion, what are the possible causes of judicialization of access to medicines?” and *(b)* “In your opinion, what are the possible consequences of judicialization of access to medicines?” All the interviews lasted from 30 to 45 min and were conducted by the same interviewer in Spanish or Portuguese. The interviews were audio-recorded and transcribed verbatim. To guarantee the quality of the data, all the transcriptions were sent to each participant for checking and correction. In order to maintain the participants’ confidentiality, only the country name and the represented stakeholder were informed in the results. All participants signed informed consent before the interview.

**Fig. 1:**
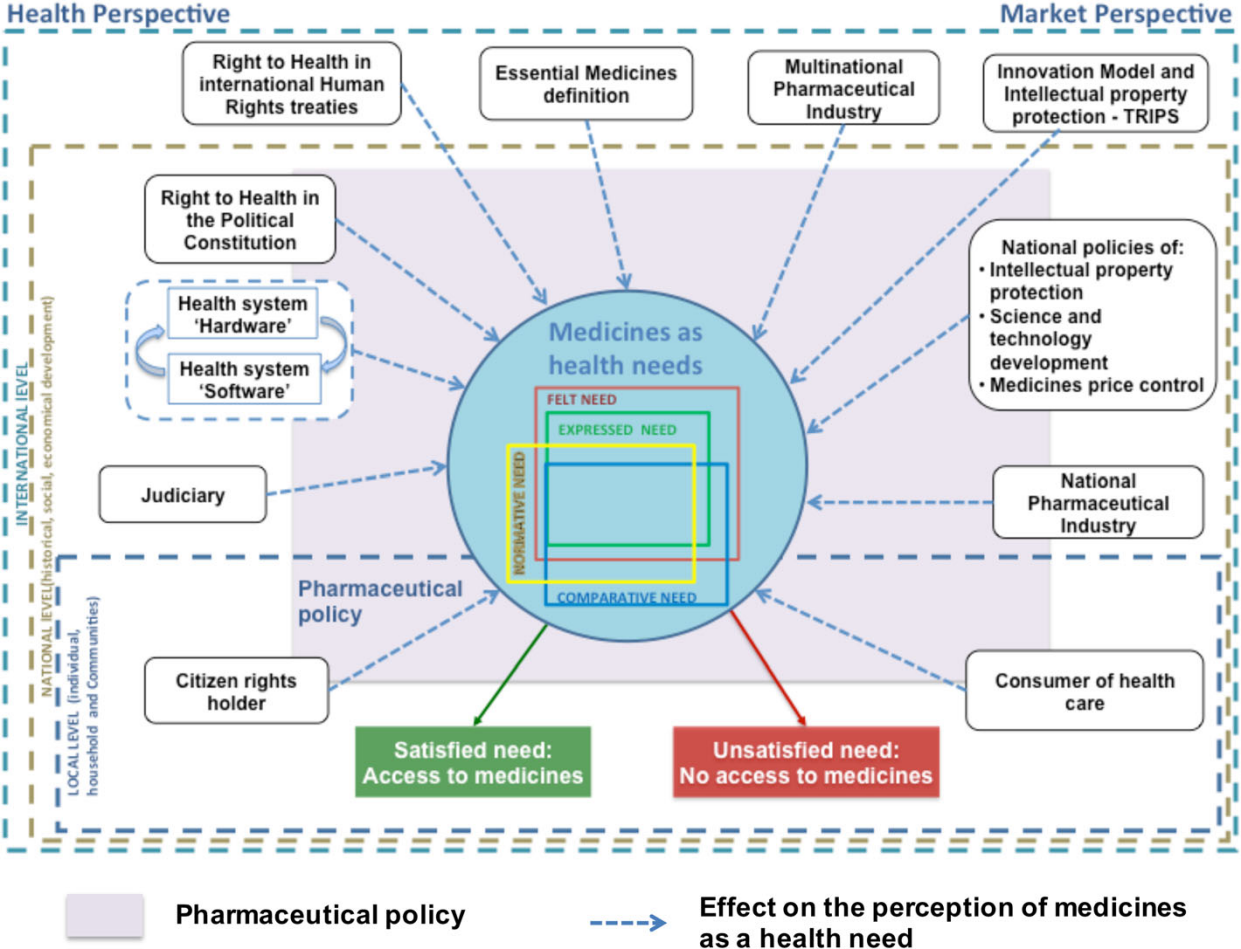
Theoretical model for medicines as health needs. The theoretical model comprises stakeholders, policies and practices that modulate the perception of medicines as a health need from two perspectives - health and market - at three levels: international, national and local levels. The different perceptions created of medicines as a health need (according to Brashaw’s categories) do not always coincide, and as a result of this “conflict” the patients do not get access to the medicines they perceive as a need. The health system scheme ('software' and 'hardware') was adapted from Sheikh et al. (2011). Pharmaceutical policy is represented as a square behind the stakeholders and policies considered at the national level, since pharmaceutical policies could adopt different forms according to the context: (**a**) a unique document considering all the aspects defining them; or (**b**) a policy that guides the development of the other policies. Source: Vargas-Peláez, et al., 2017 [1] with permission of the Journal Social Science and Medicine

Thematic analysis was applied to the verbatim using a framework approach, following the methodology proposed by Pope et al. [9]: familiarization with the data, identification of the thematic framework, indexation, charting and mapping and interpretation. The theoretical model for medicines as health need [1] was adopted as framework. The categories for the analysis were previously adopted, and correspond to the elements (stakeholders and policies) considered in the theoretical model (Fig. 1). Two researchers (CMVP and MRMR) conducted the analysis manually. In case of disagreement the reviewers discuss and if consensus was no reached, a third person made the decision (MRF) on the final code. The data from interviews were sorted by country and stakeholder in order to identify similarities and differences in their perceptions on the causes and consequences of judicialization of access to medicines. The verbatim of the selected segments of speech in the original language can be found in the Additional file 2.

## Results

Representatives from Argentina, Brazil and Colombia considered judicialization of access to medicines as a widespread phenomenon in their respective countries, while in Chile the respondents highlighted that most lawsuits related to the right to health were filed against the private insurers (ISAPREs) because of unjustified increases in the insurance premiums.

The causes and consequences of judicialization of access to medicines mentioned by the stakeholders’ representatives were related to aspects from both perspectives considered in the theoretical model: health and market. Below, the possible causes and consequences mentioned are presented by level (international, national and local level) according to the categories considered in the theoretical model.

### International level

At the international level, only Colombian representatives highlighted the conflict between market and human rights, and the global dominance of an economistic view of the fundamental rights, which prevent access to medicines. In addition, the interest and lobby of the pharmaceutical industry for both the recognition of health as a fundamental right and the adoption of the third payer model in the national health systems, were also recognized as a factor that contribute to litigation, since these policies can guarantee market and financing of new and expensive medicines (Table 4).

**Table 4:** Examples of quotes for the categories at International level

Category	Causes
Right to health in the International Human Right treaties and essential medicines definition	“… We all know that Big Pharma has been the most important lobbyist for pushing UN, WHO, everyone, to make the right to health a fundamental right in all countries, as it was clear [for pharmaceutical industry] that the people individually would not be able to buy and pay for the costs of their products, and the best thing about it was that the states have to pay for [the medicines]” (Colombia, Patient).
The market and the Innovation model and intellectual property protection – TRIPS	“I think that [judicialization] is closely related to the R&D model; and to how the pharmaceutical industry resolved the price issue very easily by means of what would be called third-party payer models. Thus, for them, it is no longer a problem that medicines may cost COP 600 million pesos or COP 700 million pesos patient/year, because in the end it is not the patient himself that pays, but the [health] system” (Colombia, professional).

According to the theoretical model adopted, from the market’s perspective, two factors were recognized as the main causes of judicialization of access to medicines: the emergence of expensive technologies and the pharmaceutical industry’s interest for profit. For the respondents, new medicines create huge expectations about their impact on health, even in cases where they actually do not represent an additional therapeutic value (failure of the current innovation model). The aforementioned aspects were highlighted by the majority of the stakeholders’ representatives from the four countries, but to a lesser extent the Judiciary’s ones. Only representatives from Colombia mentioned the relationship between the Agreement on Trade-Related Aspects of Intellectual Property Rights (TRIPS) (patent protection), medicines’ high pricing and judicialization of access to medicines (Table 4). Consequences were not mentioned at this level.

### National level

#### The right to health in the political constitution

In this category three aspects were mentioned as causes of judicialization: (a) the Constitution’s broad definition of the right to health (e.g. Argentina and Brazil); (b) the creation of judicial mechanisms to protect the right to health (Colombia); and (c) the government decentralization where the population’s health is a Provincial rather than a National Government’s responsibility (Argentina) (Additional file 3: Table S1).

Stakeholders from Argentina, Chile and Colombia mentioned consequences of judicialization relative to the national recognition of the right to health. The negative consequences included the *pharmaceuticalization* of the right to health, and the inappropriate interpretation of the right to health as an unlimited and individual right. The respondents pointed out two positive consequences. First, the visibility of the right to health as an issue relevant to the policy resulted from the media impact towards the judicial cases. Second, the generation of jurisprudence related to the right to health, which in the Colombian context resulted in the recognition of the right to health as a fundamental right, but in some way ignoring the collective dimension of this right (Additional file 3: Table S1).

#### The health system

##### ‘Hardware’

Taking the organization of the health systems as a cause of litigation, the respondents mentioned aspects that result in barriers to access to healthcare services timely. Such aspects include the health systems’ fragmentation, the health systems’ decentralization without adequate coordination, inefficiency of health system managers, inappropriate organization of the healthcare service networks, and limited availability of human resources, particularly specialized health professionals.

The lack of a consolidated Health Technology Assessment (HTA) agency (in Colombia and Argentina) and the limited financial resources of the health systems to include new, expensive medicines in the coverage were pointed out as an important cause of judicialization of access to medicines (Additional file 3: Table S1).

The respondents from Argentina, Brazil and Colombia mentioned some negative effects of judicialization of access to medicines on the health systems’ ‘hardware’. First, litigation prompts the creation of additional administrative processes to meet the judicial demands. So, the health system’s operating costs increase and the management becomes more complex, generating inefficiency. As a result, the timely access to healthcare and medicines by the people that do not use the judicial pathway is compromised.

Second, some lawsuits favour the public financing of medicines without evidence of efficacy, safety or cost-effectiveness. In these cases there are two possible scenarios: (a) the health system’s resources focus on financing expensive medicines with poor therapeutic value versus the medicines covered by the health system; or (b) lawsuits favour the treatment of a limited number of patients (e.g. rare diseases) at the expense of access to essential medicines for the rest of the population. According to representatives of the health professional organizations from Brazil and Colombia, both scenarios may compromise the long-term sustainability of the health systems.

In this sense, in all the studied countries, the health system managers’ representatives remarked that the diversion of resources for financing the medicines covered by the health system to the financing of uncovered medicines accessed through litigation compromises their liquidity. This happens in Brazil and Chile because the managers do not receive any reimbursement for the uncovered medicines. In Argentina and Colombia, this is a result of the belated reimbursement from the SUR or the Solidarity and Guarantees Fund (FOSYGA by its name in Spanish), respectively. Additionally, sometimes the judicial decision also causes resource diversion among the health system’s subsectors, having a regressive effect mainly when the public sector must finance medicines for patients treated in the private sector in Brazil and Argentina.

Other negative impacts mentioned include the healthcare fragmentation when the lawsuit requires only the medicines but no other services needed for a comprehensive care (as stated by a Colombian patient organization’s representative). A Brazilian health system managers’ representative also stated difficulties in guaranteeing the quality of the products since the medicines required by lawsuits have a different logistics process with poor quality control (Additional file 3: Table S1).

##### ‘Software’

Representatives of the four countries highlighted two aspects related to the health systems’ ‘software’ as causes of judicialization: (a) the weakness of the states in guaranteeing the fundamental rights of their citizens; and (b) the inertia of the states for taking measures to improve access to medicines timely.

The configuration of the health system coverage was also mentioned as a contributing factor to litigation for access to medicines although the perceptions varied across the countries. In Colombia and Argentina the lack of clarity of the medicines lists (*grey zones*); the inflexibility of the medicines list and the clinical guidelines were mentioned as possible causes. In Brazil and Argentina, the health system manager representatives remarked that the differences among the health systems’ subsectors contributed to the rise of litigation in these countries.

In Chile, according to the NGO’s respondents, the explicit coverage of a limited number of pathologies and the non-existence of an explicit medicines list has influenced lawsuits filing for access to medicines. In contrast, representatives of the Executive and the health system managers in Argentina mentioned that litigation rose because the medicines coverage is not linked to a specific pathologies list.

The use of HTA criteria for defining the list of covered medicines and clinical guidelines was also questioned by stakeholders, such as, health professional organizations, patient organizations and the Judiciary in the four countries. For the respondents, the accuracy of the HTA process is not good, once the decision-makers do not have consistent data about the real demand for the treatments; usually the treatment costs are considered to be more relevant than the outcomes; and only the prolongation of life is considered as an acceptable outcome, disregarding the importance of the improvement of the patient’s quality of life.

The lack of an administrative pathway to guarantee access to uncovered medicines (needed exceptionally) in the reform of the health systems in the 1990’s was cited as factor that contributed to the origin of litigation in Colombia and Argentina in the case of access to HIV treatment. Nevertheless, the later establishment of reimbursement for uncovered medicines, without medicine price control, guidelines and mechanisms to guarantee transparency, were mentioned as contributing factors to litigation for access to medicines.

Representatives from Colombia, Chile and Argentina emphasized that privatization of healthcare facilities, introduction of market logic in the health system, and poor capacity of the state to oversee and to impose sanctions to the health system’s stakeholders are among the main causes of litigation in their countries. As a result of the aforementioned factors, the organization of the health systems itself lead the health system managers deny the provision of covered services and medicines, especially the more expensive ones, in order to earn profit. In contrast, especially health system managers’ representatives from Argentina, Colombia and Chile mentioned that the people do not consider them as legitimate stakeholders. Thus when the health system manager denies some medicine, the people think that it is because the manager wants to make more profit, but not because the medicine is not the best option for the patient.

Regarding prescription practices, for representatives of the physician organizations and patient organizations, the questioning about the medical prescription by the health system causes litigation because it is the prescriber who better knows the patient’s clinical situation and has the information to decide the best treatment option. On the other hand, for representatives of Argentina and Brazil of Executive, health professional, and health system managers, the fact that physicians disregard the impacts of their prescription practices (off-label uses, unlicensed medicines, or medicines still in a research stage, and do not comply with the health system’s clinical guidelines) upon access to medicines for the population was considered a cause of litigation (Additional file 3: Table S1).

Few consequences of judicialization of access to medicines were mentioned by Chilean representatives as this is a recent phenomenon in the country. In Argentina, Brazil and Colombia lawsuits have made some limitations of the public policies evident, and have pressured the government to make structural positive reforms. Among these reforms, they mentioned the updating of the list of covered medicines, the creation of HTA agencies, the establishment of new mechanisms of reimbursement, and better overseeing of the health system stakeholders.

On the other hand, representatives from Colombia and Brazil mentioned some negative impacts of litigation such as the inclusion of new medicines because of their high-cost instead of their impacts on the public health. Therefore, the health policies design is highly influenced by the heath needs of few patients who are able to access the Judiciary, at the expense of the rest of the population’s health needs.

In all the studied countries, litigation was considered positive when lawsuits involved covered medicines that for some reason were not supplied to the patient, because it forces the health system managers to carry out their duties.

Nevertheless, the respondents recognized that when lawsuits require uncovered medicines by the health system, litigation jeopardizes the health system sustainability. Since the judicial decision must be fulfilled in a short time (usually 48 h), the managers lose their bargain power with the pharmaceutical industry and/or healthcare services providers. Moreover, litigation can induce or worsen the inequity in the health systems, in the cases that lawsuits are filed by patients assisted in health facilities of the private sector expecting to get quick access to the medicines in the public sector.

Furthermore, particularly in Brazil and Argentina, judges often grant injunctive relief without resolving the merits of the lawsuit, then the health system managers cannot interrupt the supply of the treatment, even if it is no longer effective.

Representatives of Colombia, Brazil and Argentina also mentioned that litigation promotes corruption, as it does not allow the proper execution of the overseeing processes. In addition, according to representatives from Colombia, litigation, rather than solving the underlying problems of the health systems, such as unequal access to health services and medicines, makes them worse and reduces the health systems’ credibility and governance (Additional file 3: Table S1).

#### Pharmaceutical marketing

The influence of the pharmaceutical industry in the occurrence of litigation was recognized in the four studied countries. The stakeholders’ representatives mentioned that the pharmaceutical industry uses lawsuits as a way to pressure the health systems for financing new medicines or branded medicines.

The interviewees mentioned some strategies by pharmaceutical industry to prompt litigation for access to medicines, including the relationship with prescribers, patient organizations and lawyers and marketing campaigns to discredit generic medicines, which results in the requirement of specific brands both by prescribers and patients.

With regard to consequences, Argentinian and Colombian representatives remarked that the pharmaceutical industry is the stakeholder that profits mostly from litigation for access to medicines, since the financing of their products becomes guaranteed (Additional file 3: Table S1).

In addition, Argentinian Executive’s and NGO’s representatives and Colombian health system managers’ representatives mentioned that the lack of information about medicines, financially or intellectually independent of the pharmaceutical industry, also contributes to litigation for access to medicines.

#### National policies for science and technology development, intellectual property protection and medicines prices control

The weakness of national research and development (R&D) policies and the lack of medicine pricing control were recognized as causes of litigation for access to medicines in Colombia (patient organization and health professional organization). In contrast, the monopoly created by the patent protection was also mentioned as a possible cause of litigation in the four studied countries.

Impacts of litigation for access to medicines on these policies were mentioned in Brazil and Colombia. In Brazil, R&D policies have become interrelated with policies for access to medicines since the creation of the Specialized Component of the Pharmaceutical Assistance (CEAF). In Colombia, since 2012 the Ministry of Health has established maximum prices for uncovered medicines reimbursement from the FOSYGA to the EPS (Additional file 3: Table S1).

#### Judiciary

In general, representatives of all stakeholders, except the patient organizations, mentioned causes of judicialization related to the Judiciary. In this sense, the respondents called attention to the judges’ limited technical knowledge about medicines and their vision favouring the supply of the required medicine as always the best alternative for the patient.

Other aspects pointed out as possible causes of litigation included the judges’ perception that the medical prescription is a sufficient technical support for granting access to the medicines required through lawsuits, the judges’ unawareness of possible conflicts of interest in which the prescriber could be involved, and the judges’ non-recognition of the health insurers’ technical arguments.

According to Argentinian and Colombian representatives, litigation is the result of the judges’ awareness on the right to health (Executive and NGO). Brazilian and Colombian representatives also attributed this to the judges’ wide interpretation of the right to health, considering it as an unlimited right and that any measure aiming to limit it constitutes a violation. This perception might be a consequence of the judges’ unconsciousness of the impact of their decisions on access to health services by the general population.

The aforementioned aspects were also cited by the Chilean patient organization’s representative, who did not consider them as a negative aspect; in contrast to the other participants.

Particularly in Colombia, an NGO’s representative remarked that litigation occurs in the country because the judges conceive that granting access to medicines by means of individual lawsuits is a progressist action and denying it is a neoliberal one.

Chilean and Brazilian representatives highlighted as a cause of litigation the fact that the judges do not consider the health system’ regulation and organization in their decision-making process. However, for a Brazilian Judiciary’s representative, this non-recognition is justified when the health system’s regulation does not guarantee access to medicines to the population and infringes the right to health.

Other causes of judicialization of access to medicines related to the Judiciary mentioned include the easy access to the justice; and the judges’ position of always favouring the patients, seen as the *weaker party* involved in the lawsuits, promotes judicialization (Argentina, Colombia and Chile; manager, Judiciary, NGO) (Additional file 3: Table S1).

In general, the cited impact of judicialization of access to medicines on the Judiciary were negative. They include the additional expenses and the overcharge that compromise the Judiciary’s responsiveness (Executive, managers and health professional organizations, representatives of the patient organization, Argentina, Colombia).

The loss of effectiveness of lawsuits to guarantee access to medicines, resulting from the large number of judicial cases was also cited as a negative impact of litigation in Brazil and Colombia, but with two different approaches. On one hand for a Brazilian health system managers’ representative such loss results from the overcharge of the health system managers, which reduces the manager’s responsiveness. On the other hand for the Colombian patient organization’s representative this loss is related to the inefficient punishment of health system managers when they do not comply with a court decision.

The Judiciary’s representative considered as positive impact the judicial protection of the right to health from omissions of the Executive and the Legislature, and the recognition of this fact by the population. In contrast, the health system managers’ representative considered that the judicial intervention and the wide interpretation of the right to health result in abuses by the people who aim to get access to products and services, which are not considered healthcare, most of them related to the right to free development of the personality.

In the Brazilian context, one of the positive impacts of judicialization was the creation of technical teams to assist in the judges’ decision-making (Additional file 3: Table S1).

### The local level

According to representatives from Argentina, Colombia, and Brazil, lawsuits for access to medicines occur because the patients nowadays have more access to information and can, for example, press the physician to get a certain product that he or she has seen on the Internet. In this sense, some respondents remarked that most of the currently available information about healthcare products is of poor quality (Argentina, Colombia and Brazil; Executive, managers, professional, NGO).

For Argentinian representatives, lawsuits result from the non-use of administrative pathways offered by the health system to guarantee access to medicines in case the health system manager does not comply with its obligations (Executive, Managers, and NGO). While Colombian representatives considered that most lawsuits for medicines included in the benefits plan occur because the users do not know what medicines are covered by the health system (Executive, health professional organization).

In Argentina, according to a Judiciary’s representative, litigation for access to medicines occurs because the citizens recognize this branch as ‘the saviour’, while an Executive’s representative considered that lawsuits for access to medicines are filed because now the citizens are more aware of their right to health. In Colombia, respondents mentioned that the creation of patient organizations and the unwillingness to pay of the higher income population also have an important influence on litigation for access to medicines (NGO).

In Brazil, health system managers’ representatives stated that one of the causes of judicialization was the organization of the civil society in 1990 to require access to HIV treatment, which at that time was expensive. In addition, they mentioned that the people are now unwilling to change their lifestyle and expect that all their health problems are resolved by medicines (Additional file 3: Table S2).

In the four countries, the respondents considered that a positive effect of litigation for access to medicines is that lawsuits defend the right to health of the population and the patient gets access to the medicines required. Nevertheless, the respondents also noted that judicialization has negative impacts when the Judiciary concedes to the patients medicines without evidence of efficacy or safety or medicines that should be used in advanced stages of the disease. In such cases, the patients are exposed to unnecessary risk to their health or the therapeutic alternatives are prematurely used up.

In all the studied countries, the respondents highlighted that litigation is an unequal solution to the barriers to access to medicines, because lawsuits have an individual scope and access to both the health services and the justice depends on the individual’s socioeconomic characteristics. Thus, judicialization of access to medicines ends up benefiting the people with higher income and/or who are more empowered. They have more possibilities of creating patient organizations to claim their rights in the court. In this way, representatives emphasized that an individual who benefits from a lawsuit jumps the queue and passes over those who must follow the administrative pathway.

In Colombia, the most relevant positive effect of judicialization of access to medicines is raising awareness of the right to health. However the representative also recognized that, due to the huge number of lawsuits, nowadays litigation is no longer an efficient pathway to obtain access to medicines and sometimes it ends up delaying the user’s access to healthcare.

Finally, another negative effect of judicialization of access to medicines is the social movement fragmentation into those pro-branded medicines and pro generic medicines (Additional file 3: Table S2).

## Discussion

This study analyses factors that influence the occurrence and consequences of judicialization of access to medicines in Argentina, Brazil, Chile and Colombia from the perspective of the stakeholders involved in this phenomenon, using a previously developed framework. Since this theoretical model allows a broad view of the factors involved, it is useful to conduct a cross-country analysis of this phenomenon.

The comparative analysis showed that in Brazil, Colombia and Argentina, judicialization of access to medicines is more common than in Chile. This result coincides with the findings of previous analyses [5, 10–12]. These differences can be explained by two factors: the recent change on the axis of the Chilean Constitutional Tribunal for interpreting the right to health [13] and the late establishment of an explicit (or positive) list of coverage of medicines in the health system.

In the first case, while the Judiciary from Argentina, Brazil and Colombia, was sensible to lawsuits of patients with HIV that claim access to antiretroviral medicines in the 1990s, the Chilean Courts did not concede access to these medicines. Indeed, the earliest successful court cases involving access to medicines and health services occurred only at the end of the 2000s in this country [14], when the Supreme tribunal moved from an “individualistic/contractual” interpretation of the right to health to a “social perspective” one, that conceptualizes health as a social right [13].

Similarly, in the case of the explicit medicines list, the Explicit Guarantees in Health (GES) were established in 2004 [15], which was much later in comparison with the other three countries, which established explicit lists in the 1990s [16–18].

The analysis also showed that the aspects of the international level, recognition of the right to health in human rights treaties and the TRIPS agreement, are poorly recognized by the stakeholders, with the exception of some representatives from Colombia. The disregard of the aforementioned factors, especially TRIPS agreement implementation, neglects to a certain extent the effects that the crisis of the current innovation model has on the occurrence of judicialization of access to medicines.

At the national level it was observed that judicialization of access to medicines emerged in Argentina, Brazil, Chile and Colombia regardless of aspects such as the recognition of the right to health in the constitution or the proportion of population covered by the public and private sectors. Although all the countries have signed human rights treaties [19], only in Brazil health was explicitly considered a fundamental right during conduct of the interviews in this study [20]. In the second case, in both Chile and Brazil most of population depend on the public sector for accessing healthcare services.

In the four studied countries, common causes were identified such as the health systems’ limitations in guaranteeing universal, equitable and timely access to health services and medicines covered by the health systems. According to the representatives, these limitations are related to management inefficiencies, health services networks fragmentation, stakeholders’ corruption and weak state regulation capacity, which would result in loss of credibility in the health system.

The representatives’ responses showed that the measures to regularly update the lists are insufficient for controlling litigation for access to medicines. Notwithstanding, the combination of the health system’s lack of credibility and the expectations created by the pharmaceutical marketing about the medicine outcomes makes the discussion about the judicialization causes concentrate on the comprehensiveness of the list of covered medicines.

In fact, our results show that the pharmaceutical industry, by means of the marketing practices, reinforces the *“fallacy of the out-dated medication list”* and challenges the HTA criteria. In this way the discussions about medicines inclusion in the health system focus on the premise of “the health system does not cover expensive medicines” rather than on the population criteria considered in the Health Technology Assessments.

On the other hand, the role of the physicians’ prescription practices in the occurrence of judicialization of access to medicines was recognized in the four studied countries. Nevertheless, strategies to counter the pharmaceutical marketing or to make the relationship between the pharmaceutical industry and prescribers transparent were not cited by the respondents. These strategies could contribute to reduce the frequency of lawsuits claiming uncovered medicines [21].

Furthermore, the responses of the participants show that the question “why the new medicines are that expensive?” as well as the relationship among the public policies for guaranteeing access to medicines and the intellectual property protection and the science and technological development policies are aspects frequently neglected in the discussion of litigation of access to medicines.

The disregard of some sectors for these political economy features of the right to health, had been previously described in Colombia [22], and our result suggest a similar situation in Brazil, Argentina and Chile. In addition, this neglect compromises the ability of countries to create strategies at a national and a regional level aiming to defend their health sovereignty against the pharmaceutical industry’s abuses.

In the case of the Judiciary, the aforementioned aspects influence their decision-making. Although the judges’ desire is to promptly protect the patients’ right to health, they usually do not recognize that their decisions can have adverse effects, especially when uncovered medicines are granted. This comes from the fact that the health system manager has a short time to respond, and needs to use exceptional pathways for procurement, which are usually difficult to control, favouring diversion of resources and corruption.

Furthermore, the Judiciary’s representatives argued that it is ethically unacceptable to *sacrifice the individual right to health* in order to protect the collective right to health. This evidenced the conflict of this view with the Executive’ and health system managers’ perspective that consider that what is actually unethical is to *sacrifice the collective right to health* in order to protect the individual right to health. In addition, they argued that the exposure of the patient to unsafe or unnecessary medicines, indeed, constitutes a violation of the right to health.

As regards the consequences of judicialization of access to medicines, our results coincide with the impacts reported in other studies [3]. Particularly our study showed the impacts related to inequality induced by litigation in the distribution of the health system resources. In the present study, the respondents recognized that litigation is an unequal solution to access to medicines, because the lawsuits are individual, and access both to healthcare and justice services highly depend on the socioeconomic characteristics of the population. Moreover, it is possible to observe that those who get the access to medicines granted by the judge jump the queue and pass the people who follow official or predefined pathways of the health system.

Concerning the positive impacts of litigation, one of the most emphasized ones was the pressure that this phenomenon exerts over the health system managers to fulfil their responsibilities [7]. Nevertheless, the respondents also highlighted other impacts such as the health system’s governability loss, which can compromise the implementation of corrective measures to control litigation and to improve the health system’s performance.

Following the theoretical model, a remarkable finding of our study includes the fact that only in Brazil judicialization has had impacts over R&D policies, including measures as the establishment of public-private partnerships for technology transfer in order to locally produce the medicines considered a priority for the public health system (SUS) [23].

In Colombia, litigation also encouraged the recognition of the right to health as a fundamental right, the development of policies for controlling medicines prices, and the creation of a specific commission of the Judiciary that supervises the measures taken by the Ministry of Health to meet the commitments established by ruling T-760/2008^1^ [24].

This qualitative study does not aim to generalize its findings, but describe the perceptions of the stakeholders involved in judicialization of access to medicines. This is the reason why the variation in the number of the stakeholders’ representatives across the studied countries is not believed to have a considerable effect on the results. A limitation of this study includes the fact that most of the interviews were conducted only in the capital cities, with the exception of Brazil and Argentina. Therefore the view of stakeholders’ representatives located in places where there might be more barriers to access to the health system or the judiciary were not reached. Another limitation is the fact that pharmaceutical industry’s representatives did not accept to participate, thus the study does not consider their views.

## Conclusion

The analysis of judicialization of access to medicines using the proposed theoretical model allowed a comprehensive view of the phenomenon. In this way, our analysis explored the influence of different factors from the health and market perspective at different levels (international, national and demand-side), which can influence the occurrence of judicialization as well as the feedback effects that the phenomenon can bring.

According to the results of this study, the causes of judicialization of access to medicines commonly described by the studied countries’ representatives were those related to the organization of the health system (‘software’ and ‘hardware’). They mentioned especially issues about the definition of the list of medicines covered by the health system and the non-social legitimacy of the Executive and health system managers. The influence of the international context and the national economic and social policies on judicialization were poorly known.

The visibility of the right to health as a relevant issue for policy and pressure on the government to implement positive structural reforms were the two main positive consequences pointed out. In all the countries studied, respondents stressed that litigation is an unequal solution to barriers to access to medicines, since the processes are individual in scope and access to health services and justice depends on the socioeconomic characteristics of the individual.

Our analysis shows some similarities in the causes and consequences of litigation for access to medicines in the studied countries, despite the differences in the contexts and the possible aspects that define the extension or occurrence of this phenomenon in a specific time frame. The result suggest that this kind of analysis, applying the theoretical model adopted, creates the possibility of identifying critical points that can guide the policy making at both national and international levels to improve the performance of the health systems and control the lawsuits for access to medicines.

## Additional files

Additional file 1: Health systems descriptions. (DOCX 1112 kb)

Additional file 2: Quotes in original languages. (DOCX 29 kb)

Additional file 3: Quotes translated into English. (DOCX 27 kb)

## Abbreviations

CAEC: Additional Coverage for Catastrophic Diseases - Cobertura Adicional para Enfermedades Catastróficas
CEAF: Specialized Component of the Pharmaceutical Assistance – Componente Especializado da Assistência Farmacêutica
CONITEC: National Commission for Incorporation of Technologies in the SUS - Comissão Nacional de Incorporação de Tecnologias no SUS
EPSs: Health Promoting Companies – Empresas Promotoras de Salud
FONASA: National Fund of Health – Fondo Nacional de Salud
FOSYGA: Solidarity and guarantees fund – Fondo de Solidaridad y Garantía
HIV: Human Immunodeficiency Virus
HTA: Health Technology Assessment
IETS: Institute of Technological Evaluation in Health - Instituto de Evaluación Tecnológica en Salud
ISAPREs: Preventive Health Institutions – Instituciones de Salud Previsional
NGOs: Non-governmental organizations
OSs: Obras Sociales
PMO: Mandatory Medical Plan – Plan Médico Obligatorio
R&D: Research and Development
SGSSS: General Social Security in Health System – Sistema General de Seguridad Social en Salud
SUR: Unified System of Reimbursement - Sistema Único de Reintegro
SUS: Unified Health System – Sistema Único de Saúde
TRIPS: The Agreement on Trade-Related Aspects of Intellectual Property Rights

## Footnotes

1 In July 2008, the Court issued judgment T-760 of 2008, in which, after a structural analysis of the crisis in access to health, it issued a set of general orders aimed at avoiding regulatory failures with respect to plans of benefits, financial sustainability, the flow of system resources, and its coverage. The Court had as its starting point a focus on rights and the insistence on participatory processes that legitimized these changes. In addition, this intervention aimed to promote equity, the proper functioning of the system and reduce the flight to litigation as a last resort to achieve access to health [25]
