# Supplementary material for: Judicialization of access to medicines in four Latin American countries: a comparative qualitative analysis
Source: Int J Equity Health. 2019 Jun 3;18:68. doi: 10.1186/s12939-019-0960-z (PMC6545681; doi:10.1186/s12939-019-0960-z)
Supplement: Supplementary file 1 — Health systems descriptions. (DOCX 1112 kb) [file 12939_2019_960_MOESM1_ESM.docx]

# The Argentinian health system

The Argentinian health system is constituted by three subsectors. Public subsector corresponds to the public health system and the Federal Program *Incluir Salud*. Social insurance subsector correspond to *Obras sociales* (OS) and the National Institute of Social Services for Retirees and Pensioners/Integral Medical Care Plan (INSSJyP/PAMI). Private sector involves voluntary health insurance by direct payment or through the OS (1,2). According to the 2010 census, 46.4% of the population had health coverage by affiliation to an *Obra Social* (including PAMI), 10.6% had coverage by a private insurance company through OS (*deregulated*), 5.1% had voluntary private insurance (prepaid medicine); 1.8% had coverage for state health programs or plans. The other 36.1% did not have coverage in health for the other ways and for medical attention depend on the public subsector (3).

## PUBLIC SECTOR:

In the public sector each of the 23 provinces and the Autonomous City of Buenos Aires are responsible for providing healthcare services in their territories. Healthcare services are provided by public hospitals and healthcare units, which are financed with national, provincial and municipal resources. National regulation related to the health system is not binding on the provinces; this is reason why the Nation must negotiate with the provincial ministries or secretaries of health the implementation of the regulatory measures in the Federal Health Council (COFESA). In addition, the Federal Program *Incluir Salud* is a Public Health Insurance system, which guarantees access to health services for mothers of seven or more children, disabled people and adults older than 70 years Non-Contributory Pensions (PNC) holders, among other groups (4). Although it is organized from the national level and operates under the aegis of the National Ministry of Health, the implementation is provincial (5).

## SOCIAL INSURANCE SECTOR:

The social insurance is conformed for about 280 National *Obras Sociales* (regulated by laws 23.660 and 23.661), armed forces, security, university Obras sociales, and the National Institute of Social Services for Retirees and Pensioners/Integral Medical Care Plan (INSSJyP/PAMI). All of them are regulated by the National Ministry of Health and the Superintendence of healthcare services. On the other hand, the Provincial OS depend on and are regulated by the ministry of health of each Province (6,7). Since the National OS creation in 1940s until 1993, they were associated with different industrial sectors that had a monopolistic right over the formal labour force of each sector. In 1993 the deregulation of OS (8) broke the monopoly allowing that the workers could choose the insurance fund according to their preference, including the option of the private insurance companies. The deregulation also allows the OS contract private insurance companies for the management of resources and healthcare services(1,9). National social health insurance, is funded by a compulsory payroll contribution from employees (3%) and employers (6%) (6). In Argentina there are almost 300 OS, the number of beneficiaries per entity vary between 3000 and more than 1 million, almost 70% of the affiliates are concentrated in only 30 insurance funds, and the distribution of the population according to age and gender among the OS is heterogeneous (7).

PAMI is funded by a portion of the compulsory payroll of the employees, compulsory income-dependent contributions of retirees (3 to 6%) and national resources (Belló and Montekio-Becerril, 2011; PNUD, 2011). Provincial social health insurance is funded by civil servants (3%-5%) compulsory payroll contributions and provincial governments’ contributions as employers (4%-6%) (2,7).

## PRIVATE SUBSECTOR:

The private subsector is constituted by private insurance companies of diverse nature (commercial societies, civil associations, for profit or not-for profit), called *Empresas de medicina prepaga*. These companies are concentrated in the bigger cities and focused on high-income population (PNUD, 2011). Private institutions provide the healthcare services in this sector. The affiliation to a private insurance company could be by two ways: (a) by the *deregulation* mechanisms, that means, the person is affiliated to an OS that has a covenant with a private insurance company or (b) voluntary private insurance that could be made for individual or companies. In the case of *deregulation*, the OS transfers part of the compulsory payroll contributions for the insurance company and the user must pay and additional premium and co-payments to get access to the healthcare services of the insurance company. In the case of voluntary private insurance, the person or company contract the service directly with the insurance company (2).

Figure A. Health System Organization and Access to medicines pathways in Argentina


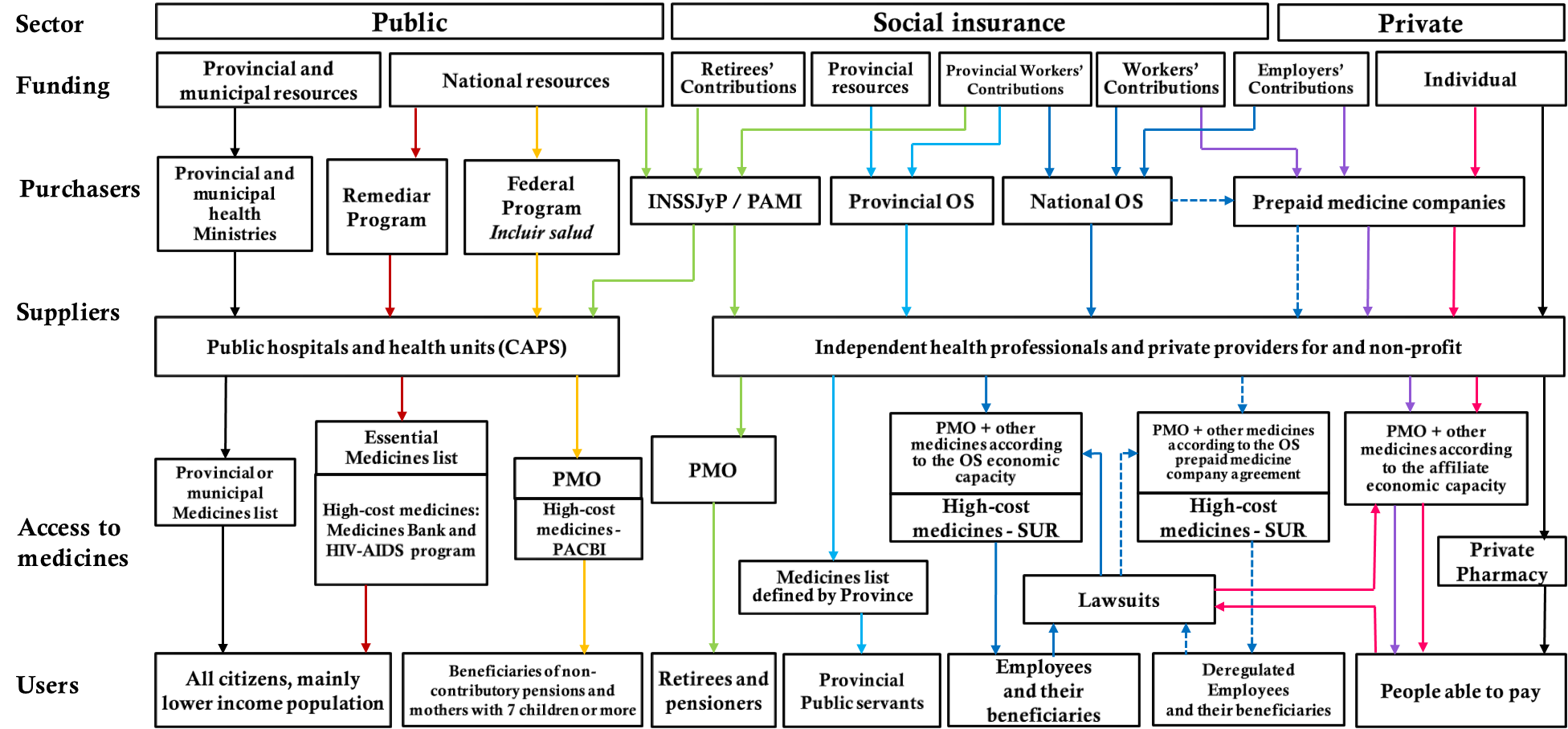


Source: Prepared by the author based on Belló and Becerril-Monteiko (2011)

# The Colombian Health system

Since 1993, two subsectors, the General System of Social Security in Health (SGSSS) and the private sector constitute the Colombian health system. The basic principles of the SGSSS are efficiency, universality, solidarity, integrity, unity and participation. The SGSSS has three regimes: contributory regime focused in formal workers and employees; subsidized regime focussed on low-income population; and special regimes focused in employees of specific sectors. People uncovered by the health system, denominated *linked* (*vinculados*), depend on the public sector for access to healthcare services (10). In 2014, 48.01% of the population was coverage for subsidize regime; 43.56% for contributory regime, 3.9% for special regimes; and 4.55% of the population was *linked* (11). In addition, the affiliates to contributory regime can buy private insurance (*medicina prepagada*).

The health system is based on the structured pluralism model (or managed competition) including market logic in the system. The social security and healthcare services networks management was decentralized and entrusted to the insurance companies, denominated *Health Promoting Enterprises (Empresas Promotoras de Salud – EPS*) which could be public, non-profit or profit companies (Vargas et al, 2002). Following the market logic, the people could choose an EPS, according with his/her preference for accessing healthcare services, and the institutions that provide healthcare services (public and private) have to compete for being contracted by the EPS (10).

## CONTRIBUTORY AND SUBSIDIZED REGIMES:

The contributory regime is funded by general taxes, oil funds, the Compulsory insurance for traffic accidents (SOAT), and contributions a compulsory payroll contribution from employees (4%) and employers (8,5%) which are collected by the EPS (12). In this regime, there are 15 EPS; but six companies concentrate 75% of the affiliates (13). The subsidized regime is funded with fiscal transfers from the national government to the departments and municipalities (*Sistema General de Participaciones-SGP*) and 1.5% of the mandatory payrolls of the contributory and special regimes affiliates. Currently there are 52 EPS in this regime, called EPS-S, 20 of them concentrate 90% of the affiliates (14). All these resources are joined in the Solidarity and Guarantee Fund (FOSYGA) and then redistributed among the EPS/EPS-S by means of the per-capita Unit (UPC) per each affiliate (15). In order to avoid adverse selection by the insurers, the UPC is adjusted for variables such as gender, age and geographical location (16). However, the subsidized regime UPC value is 12% lower than the subsidized regime UPCs value (17). Each insurer organizes the healthcare service networks with their own clinical or hospitals (maximum 30% of the value of health spending) (18) or by contracting private (profit or non-profit) healthcare service institutions (10). The EPS-S must contract with public hospitals at least 60% of health spending (18). In both of the regimes, the users must pay prorated fees and co-payments for accessing the healthcare service and some medicines covered by the health system. These values depend on the affiliate’s income and the maximum values are defined by the Ministry of Health (19).

## SPECIAL REGIMES

The special regimes are the armed forces, the National oil company (Ecopetrol) and public school and universities professors. The healthcare network as in the aforementioned regimes, could be organizing by contracting their own institutions, public or private (profit and non-profit) institutions (20).

## POOR UNINSURED POPULATION

The healthcare for poor people do not affiliated to the SGSSS is funding with resources of the SGP, by means of supply subsidies (21). The Secretaries of health of each municipality, district or department are the responsible for organizing the healthcare network, which usually is constituted by public hospitals (ESE).

## PRIVATE SECTOR:

The private sector could be divided in two parts, the private insurance (*Medicina prepagada – Prepaid medical service*) and the out of pocket expenditure. Prepaid medical service is a form within the additional health plans established by Law 100 of 1993, which contributory regime affiliates can acquire, in order to obtain optional benefits such as care for events not included in POS, or different or additional conditions of hospitality and technology (22). According to the regulations, prepaid medicine companies manage and provide care and services covered by a health plan prescribed, receiving in return the payment of an agreed regular price (23). This price is adjusted considering the gender, age, and health status of the individual (pre-existences). Finally, out of pocket expenditure occurs when the people do not have coverage by the health system or when the access to the healthcare services or medicines in the health system is not opportune (20).

Figure B. Health System Organization and Access to medicines pathways in Colombia


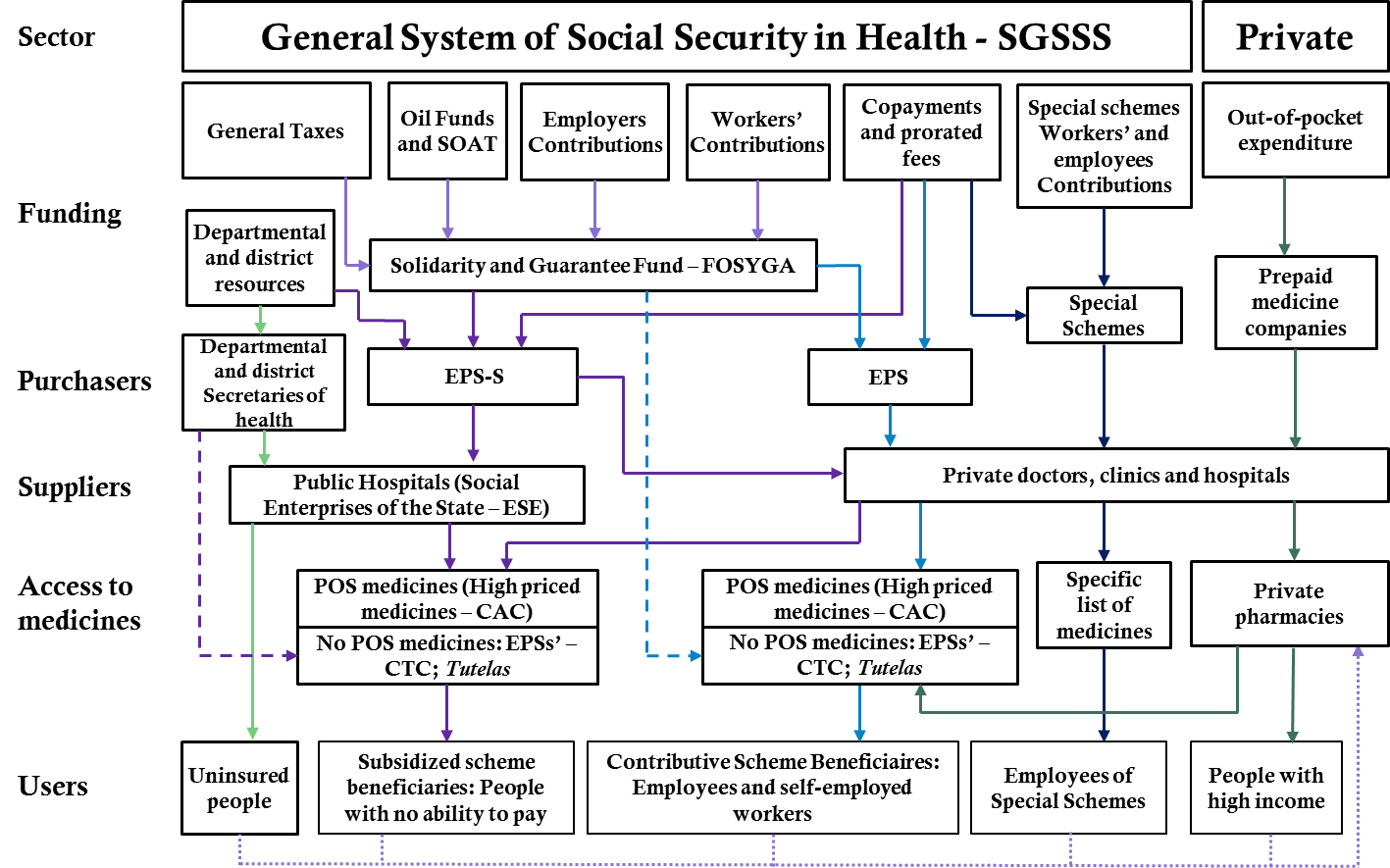


Source: Prepared by the author based on Guerrero et al (2011)

# The Brazilian health system

The health system in Brazil consists of public and private sectors. The public sector involves the Unified Health System (SUS) created by the National Constitution of 1988 considering as principles the universal and equitable access to comprehensiveness healthcare (24). The private sector, called supplementary health system, corresponds to the insurance companies and the private healthcare institutions. Although all the citizens can access the public services, in 2013, 72.1% of the population depends on the SUS for access to healthcare services and 27.9% had some health insurance plan (25).

## PUBLIC SECTOR

Since the National Constitution established the health is everyone’s right and duty of the state, all the citizens have right to access healthcare services and medicines in the SUS free of charges. The Unified Health System is funding by tax revenues and social contributions from the federal, state and municipal budgets (26). The management model of the system is decentralized and upward (from local to federal level) (27), as well as, participative by considering the voice of the deliberative bodies for social control, such as, national health conferences, health councils and intermanagerial committees bipartite (state and municipalities) and tripartite (Union, states and municipalities) (26). In addition, the health sector organization allows the government hire private healthcare services in order to complement the coverage of public health services (1).

The municipalities are responsible by the organization of the primary health care network, constituted by basic healthcare units, and the implementation of the Family Health Program (PSF). PSF works through family healthcare teams (one doctor, one nurse, one auxiliary nurse and four to six community health workers), the teams are located at PSF clinics, and each team is responsible for a specific geographical area and a defined population of 600-1000 families. To provide secondary care services the SUS is highly dependent on contracts with the private sector, especially for diagnostic and therapeutic support services. In the case of tertiary care, that include some high-cost procedures, the SUS contract these services also with private providers and public teaching hospitals. The payment of these services occurs by the modality fee for service, and the SUS define the price for each procedure (26).

## PRIVATE SECTOR

In the private sector three segments coexist: (*i*) the specific health insurance plans for public servants (civil and military) and their dependants, funded by public resources and the own beneficiaries resources; (*ii*) the health insurance plans, of elective link, funding by employers or families; and (*iii*) Autonomous private health providers, which can be reached directly by out-of-pocket expenditure (28). The three segments are funding in some way by public funds, being the most direct in the first segment. In the segments (*ii*) and (*iii*) the transference of public funds to the private sector occurs by means of tax exemptions or tax breaks that reach the households and enterprises spending with medical care and private health insurance (28,29). The insurance companies organize the healthcare service network with their own providers and by hiding other private institutions. The out-of-pocket expenditure for consultations or diagnostic procedures usually occurs when the healthcare service are not opportune in the SUS or in the health insurance, and the patient need to meet the criteria of the PCDT to receive for first time or continue the treatment (30).

Figure C. Health System Organization and Access to medicines pathways in Brazil


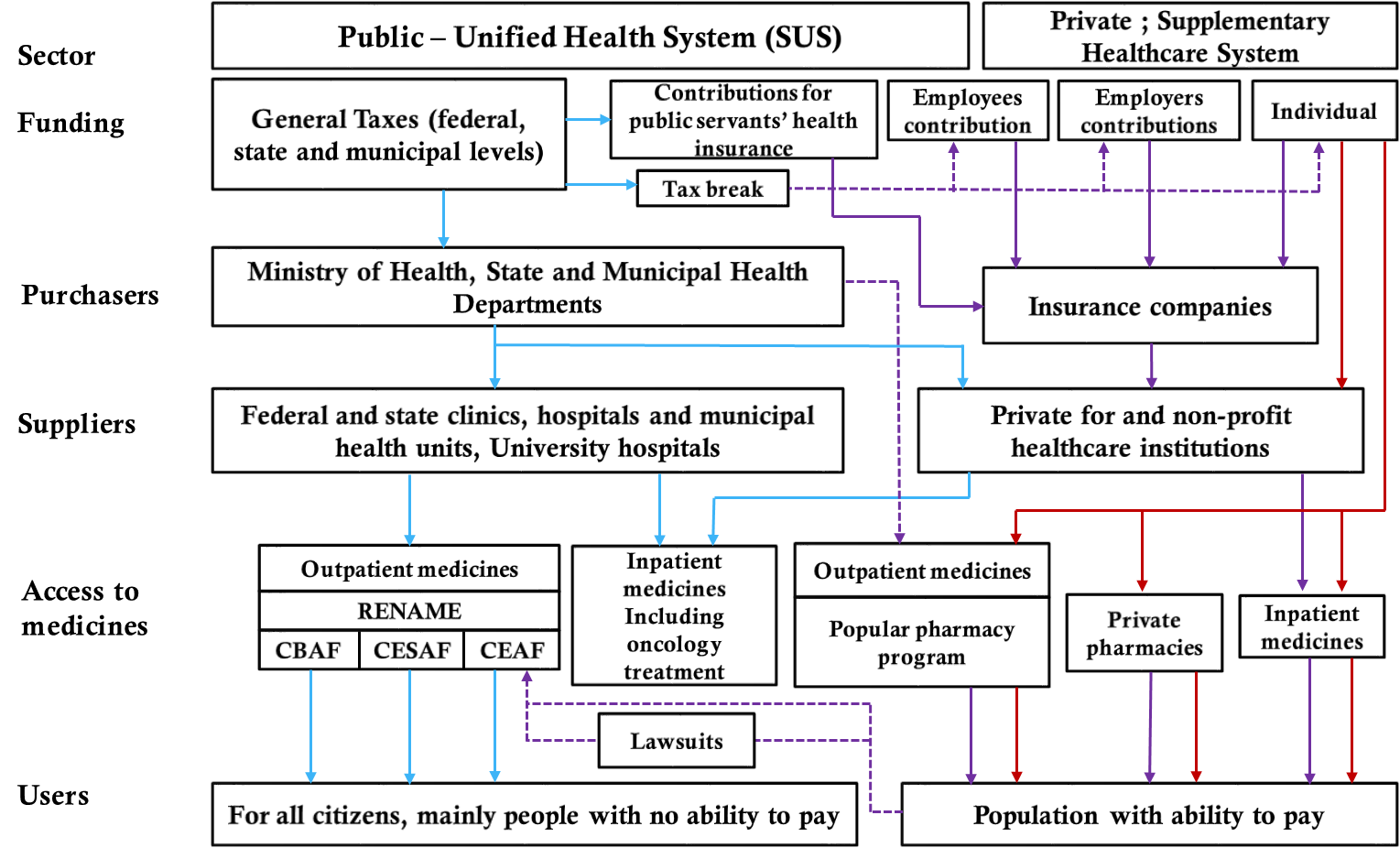


Source: Prepared by the author based on: Becerril-Montekio, *et al*. (2011)

# The Chilean health system

The Chilean health system is constituted by three subsectors. The public sector corresponds to the National Health Fund (FONASA); the private sector corresponds to the Health Insurance Institutions (ISAPREs); and the third subsector is the Armed Forces’ health system. In 2014, 77% of the population was covered by FONASA; 17% was affiliated to the ISAPRES; and the Armed Forces’ health system covered 3% (31). However, most of the resources are concentrated in the private sector, since the high-income population is affiliated to ISAPREs (32).

## PUBLIC SECTOR

The public sector is funded by general taxes, the payroll mandatory contributions of the employees (equivalent to 7% of the salary) and the co-payments, all of these resources are joined in the National Health Fund (FONASA) that constitutes a solidary system (Chile, 2014). The FONASA affiliates are classified in four groups according to their income *Group A* are people without means; *Group B* are people which income is less than one minimum wage; Group C and D are people which income is higher than a minimum wage. FONASA offers two possible modalities for accessing the healthcare services the Institutional Attention Modality (MAI) and the Free Choice Modality (MLE) (33).

## PRIVATE SECTOR

The private sector is constituted by the Health Insurance Institutions (ISAPRE). In 2015, there are 6 closed ISAPRES and 7 open ISAPREs. Closed ISAPREs belong to public enterprises (State Bank, the National Copper Corporation of Chile–COTELCO and the Chemical and Mining Society of Chile–SQM) and only affiliate their employees (31). Open ISAPREs allow registration to the entire population with ability to pay (34,35). These health plans are funding by the mandatory payroll of the employees (7% of the wage) and additional voluntary contributions to afford the total plan price. Since this insurance is individual (in contrast with FONASA that is solidary) the plan prices depend on factors such as, age, gender and health status, being cheaper for men, young and healthy people (32). In 2014, there were more than 12.000 health plans in the market (31).

## ARMED FORCES

Since 1996 the Ministry of Defence established the Armed Forces Health System that covers the Armed Forces personal and their dependents. This subsystem is funded by general taxes, and the resources are deposited in the Curative and Preventive Medicine Fund for the Armed Forces. The Armed Forces’ healthcare institutions are considered public, but usually they supply healthcare services for their beneficiaries and also sell healthcare services for other institutions in the health market. Moreover, the beneficiaries of this health subsystem also can access to healthcare services in the public services network, or even private institutions according the covenants of the Ministry of Defence (34) .

Similar to the Colombian health system, Chilean system also incorporate the market logic for the healthcare system. The individual can choose among FONASA and the ISAPREs (34).

Figure D. Health System Organization and Access to medicines pathways in Chile


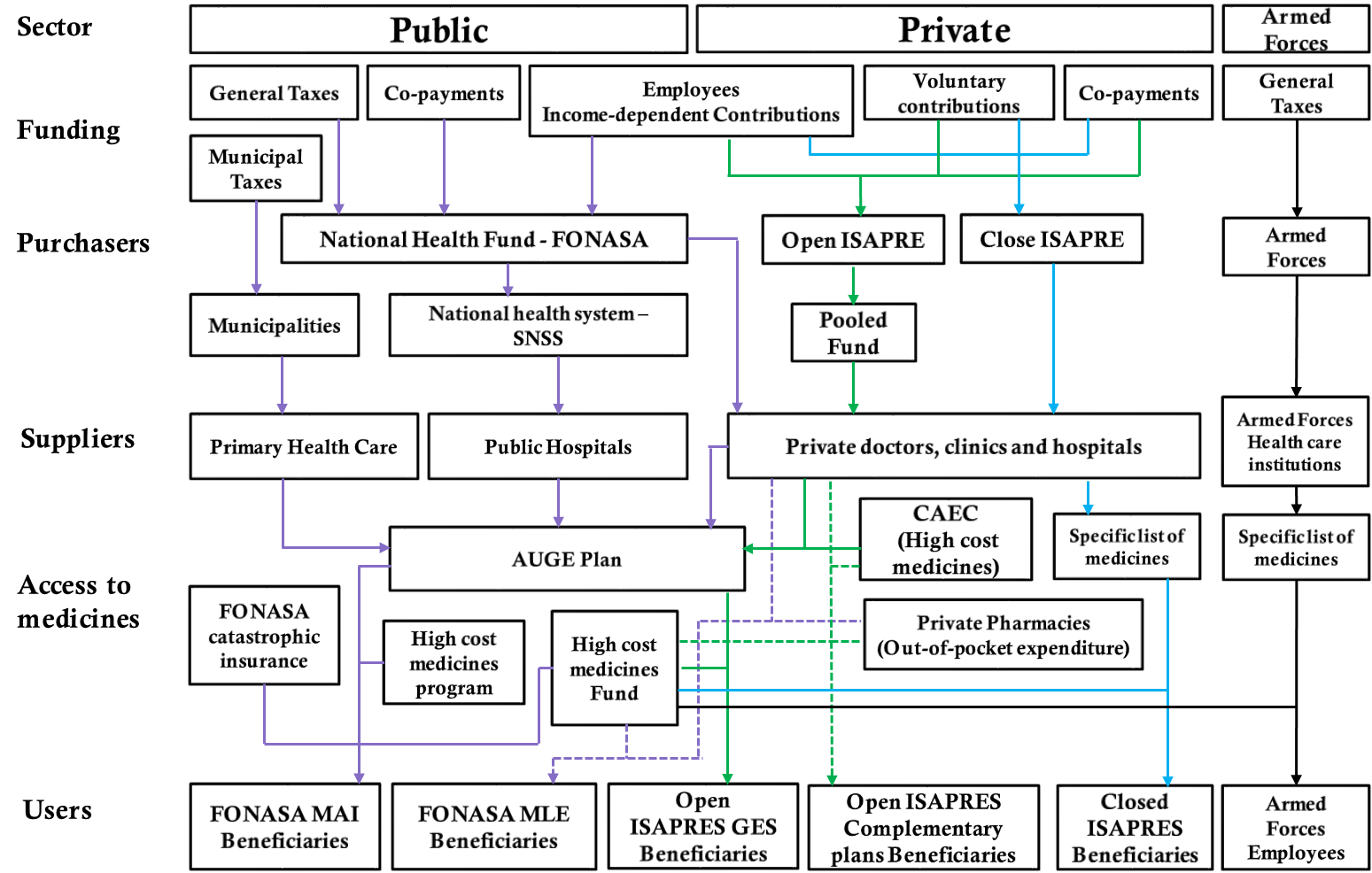


Source: Prepared by the author based on: Becerril-Montekio *et al* (2011) and Cid, *et al.* (2013)
