# Supplementary material for: Judicialization of access to medicines in four Latin American countries: a comparative qualitative analysis
Source: Int J Equity Health. 2019 Jun 3;18:68. doi: 10.1186/s12939-019-0960-z (PMC6545681; doi:10.1186/s12939-019-0960-z)
Supplement: Supplementary file 2 — Quotes in original languages. (DOCX 29 kb) [file 12939_2019_960_MOESM2_ESM.docx]

**Table S1. Examples of quotes for the categories at International level**

| **Category** | **Causes** |
| --- | --- |
| **Right to health in the International Human Right treaties and essential medicines definition** | “…todos sabemos, que las farmacéuticas fueron las que más hicieron lobby tanto en la ONU, en la OMS, en todos lados para que el derecho a la salud fuera un derecho fundamental en todos los países, porque tenían claro que la sociedad de manera individual no iba a poder comprar y pagar los costos de sus productos y lo mejor era que los Estados los pagaran” (Colombia, Patient). |
| **The market and the Innovation model and intellectual property protection – TRIPS** | “Creo que eso [la judicialización] está muy relacionado con el modelo de investigación y desarrollo, con que para la industria farmacéutica el tema del precio se solucionó muy fácil encontrando lo que se denominaría modelos de tercer pagador, entonces para ellos ya no es un problema que el medicamento valga 600 millones de pesos o 700 millones de pesos paciente/año porque en últimas no es el paciente de su propio bolsillo quien tiene que acceder [pagar], sino que es el sistema [de salud]” (Colombia, professional). |

**Table S2. Examples of causes and consequences related to the National level categories**

| **Category** | **Causes** | **Consequences** |
| --- | --- | --- |
| Right to health in the Political Constitution | “Son muchas las causas [de la judicialización]. En primer lugar, fundamentalmente, las normas tenemos que son muy amplias. Normas constitucionales y tratados con rango constitucional… que son muy amplias, en cuanto a la cobertura, con lo cual, prácticamente, cualquier paciente que pida cualquier prestación, los derechos son tan amplios que, de alguna manera, [los derechos] lo respaldan [al paciente] para pedirla [beneficios]” (Argentina, Executive).  “... [a nova constituição] era uma constituição muito mais aberta do que as anteriores, ai do regime militar, do período de repressão e uma constituição pela primeira vez na história do Brasil trazia uma série de direitos, entre eles ai, o chamado artigo 196 da constituição, que é um artigo que, de uma maneira muito ampla, muito genérica, ele prevê a saúde como um direito do cidadão e obrigação do estado, sem delimitar isso claramente” (Brazil, manager). | “Pero yo diría que uno de los aspectos más negativos [de la judicialización] es [...] lo que yo llamo [...] la farmaceuticalización del derecho a la salud, en donde pareciera que todo se resuelve con fármacos” (Colombia, University lecturer)  “La tutela es […] por el medicamento que puede ser que sea muy costoso frente a uno que está en el POS y le agrega muy poquito a la vida. O sea, la relación costo-efectividad es muy bajita pero es la persona, el derecho es individual y no colectivo. Entonces estamos presionando por cosas de altísimo costo, de bajo impacto en la salud colectiva, y la platica se nos está yendo allá. O sea, hay toda una discusión del derecho colectivo vs. el derecho individual” (Colombia, manager). |
| Right to health in the Political Constitution (cont.) | “La Constitución del 91 hizo dos cosas fundamentales en el país, introdujo el mercado en la prestación de servicios sociales, de servicios públicos en el país y en contrapeso, garantizó los derechos... [la Constitución] dejó explícitos los derechos de los ciudadanos y creo el mecanismo de la tutela para reclamarlos en caso de... de que se considerara que se violaban esos derechos” (Colombia, NGO). | “Si no tiene plata para contratar a un abogado, salen a los diarios, a los medios de comunicación denunciar una situación, y la gente no acepta ser discriminada por ser pobre, por no tener recursos, porque el estado tiene que resolverles los problemas” (Chile, lawyer). |
| Health system hardware | ... el problema… es más económico, porque los recursos son escasos. Entonces… la falta de protección… es un común denominador (Argentina, Executive)  [se debe diferenciar] Qué parte de ese problema [judicialización] es ineficiencia de la EPS, mala prestación y mala gestión de la EPS, y qué parte es un problema estructural de déficit de recurso humano, sobretodo en especialistas y subespecialistas (Colombia, manager).  Aquí [en Argentina], hay una Administración Nacional de la época de [presidente] Menen, que es el ANMAT, la Administración Nacional de Medicamentos, Alimentos y Tecnologías, que dice, cuando se cumplimenten determinadas normas, si un medicamento ...puede entrar al mercado o no. Pero no hay nadie, no hay ninguna organización nacional, que diga si eso lo va a cubrir o no la seguridad social o el Estado, está bien? No hay nada. O sea, que está al libre albedrío de cualquier médico, porque el médico puede recetar lo que quiera (Argentina, NGO). | “Mirá, a veces [los impactos] sobre la obra social, lo malo es que [los jueces] te obligan a dar una prestación, económicamente a la obra social, obviamente, no le conviene, porque por estar obligados a brindar una determinada parte de la prestación, termina dando el doble o el triple de lo que tenías que dar... a la empresa [las acciones judiciales] le perjudica porque gastas el doble de lo que tenías pensado gastar para cubrir esa enfermedad” (Argentina, Manager).  ““Do ponto de vista do estado a judicialização, ela traz uma desorganização de serviço na prática. Nós temos muita dificuldade em lidar como o volume das ações judiciais aqui no estado... Nós temos hoje uma concentração de ações judiciais aqui; e isso do ponto de vista da Secretaria estadual e das Secretarias municipais de saúde representa um volume improcessável, a gente não consegue dar respostas ao volume de ações judiciais que nós temos aqui. A estrutura do estado não está dimensionada para isso” (Brazil, manager). |
| Health system hardware (cont.) |  | “Definitivamente hay un impacto negativo dentro de las finanzas del sistema, porque al ser desordenada la forma en que la gente accede a las prestaciones no contenidas en el plan de beneficios, el sistema se ve en la necesidad de gastar una gran cantidad de recursos... en unas tecnologías terapéuticas, incluidos medicamentos.... que son muy altos y que hacen que el sistema pues tenga que dedicar gran parte de recursos” (Colombia, professional).  “Debe haber un organismo estatal que pueda surtir de estos medicamentos de alto costo, con un presupuesto dado en la parte de presupuesto fiscal porque... verás que para una entidad privada, sobretodo como nosotros que no perseguimos fines de lucro.... nosotros con una de estas sanciones [decisiones judiciales] nos vamos para atrás porque de estas resoluciones judiciales para nosotros gastar 20 millones en un medicamento, en una persona es mucha plata dentro de nuestro presupuesto que siempre tiene que tender al equilibrio” (Chile, manager). |
| Health system software | “El segundo de ellos [causas] es un incentivo perverso que se genera en la creación de las EPS en Colombia. El incentivo perverso, que es el que las mata y mata la forma en que se diseñó el sistema en Colombia, es que... el ánimo de lucro de estas empresas supone que si gastan... que si dan menos servicios [...] o gastan menos dinero, pueden ganar más dinero... Entonces hay una opinión generalizada, consecuente de ese modelo de contrato, no es que las EPS siempre lo hagan por eso, pero cada vez que nieguen o no den un servicio la gente va a creer que es en función del lucro, que es para ganar más plata que le están negando el servicio” (Colombia NGO).  “A gente identificou que havia uma já na porta de entrada [do serviço de saúde] uma resistência, né? No sentido do servidor público não fazer o que ele tinha que fazer, e ai a defensoria pública [...] tinha aquela visão de que para ela se afirmar ela tinha que ajuizar, então ela estava entrando com a ação. Então... havia um movimento favorável [na defensoria], mas o servidor não queria fazer o trabalho dele, de certa forma o não fornecimento adequado... pelo município, pelo estado possibilita algum favorecimento para alguma farmácia que seja do parente do secretário de saúde ou do perfeito. (Brazil, Judiciary).  “[En el AUGE] hay patologías que estratifican en rangos de edad el acceso a medicamentos... En este caso hay algunas cosas que... ciertos casos... de tal edad a tal edad se les dan medicamentos y a los otros no [...] Entonces los accesos no son estándares para todo el mundo, entonces van a haber personas que por un año de edad o por una situación [clínica] van a quedar fuera del acceso que tiene este programa” (Chile, Patient). | [Como consecuencia de la judicialización] “La política pública está demasiado guiada por el litigio, y en el contexto macro del sistema de salud el litigio es marginal en términos de acceso [...] menos del 0,5% de las acciones en salud se acceden [...] por mecanismos judiciales” (Colombia, NGO).  “En el caso del No POS los mecanismos de contratación de servicios de salud es fee for service, [...], nadie dice nada, nadie cuestiona nada... Entonces pues desde el punto de vista de un prestador, de un proveedor [incluyendo a la industria farmacéutica] pues esa es la lógica más razonable desde el punto de vista económico, y pues está amparado por una cosa que se llama tutela en Colombia”. (Colombia, manager).  “En el caso de las prestaciones que están dentro del Programa Médico Obligatorio, ahí no cabe duda, están a cargo de las obras sociales, [...], si se llegara a judicializar algo, [...] está correcto [...]. Si el paciente no se encuentra conforme con lo que la obra social le brinda y está dentro del Programa Médico Obligatorio, está perfecto que vaya y que vía judicial solicite, porque de alguna manera hay un contrato que se quebró” (Argentina, manager).  “Então assim, um grande número de ações individuais causam uma desorganização no sistema deles [dos entes responsáveis pela organização do sistema de saúde], que eles começam se mexer. [...] Eles começam ficar tão incomodados, que aí eles efetivamente começam a fazer alguma coisa, a alterar a política pública, entendeu? Então esse é um aspecto positivo mesmo” (Brazil, Judiciary). |
| Pharmaceutical marketing | “Há evidência das indústrias farmacêuticas e os médicos aí, que são de alguma maneira influenciados pela indústria, também [os médicos] acabaram entrando nessa coisa [a judicialização] na medida que perceberam que o judiciário tinha, digamos assim, bons olhos para esse tipo de coisa [judicialização]” (Brazil, manager).  “Pero también hay que ver que hay intereses creados detrás de ellos [organizaciones de pacientes], que provocaron una judicialización también, y todavía importante ¿no? O sea se mueven los laboratorios muchas veces detrás de los pacientes y hacen que bueno, rápidamente un medicamento que salió nuevo, ya al otro día lo están pidiendo. Decís ¿pero cómo puede ser? ¿no? Claro, el laboratorio se quiere resarcir rápido de los gastos de la investigación, y ya lo quiere colocar [el medicamento] en el mercado (Argentina NGO).  Las transnacionales [farmacéuticas] [...] van a presionar por vender los medicamentos de alto costo siempre y usa todas las estrategias para ello, desde las asociaciones de pacientes hasta los mismos abogados pagados para las asociaciones de pacientes para que exijan los medicamentos. Entonces tienen las transnacionales empujando la tutela (Colombia, NGO). | “Unos datos chéveres, que teníamos, mostraban cómo unas farmacéuticas se llevaban, concentraban todos unos rubros y recobros buenísimos, súper jugosos, o sea, claramente había unas empresas a las que sí les iba muy bien con los recobros” (Colombia NGO). |
| National policies for science and technology development, intellectual property protection and medicines prices control | “Cuando [la innovación] está en manos privadas, no podemos saber cuánto es el valor de esa innovación [...] el interés por el lucro se interpone en medio del acceso, y de alguna manera [...] se sujeta a estos países [subdesarrollados] a la regla del mercado, es decir, proteger patentes [...] que nosotros entendemos que es un estímulo para [investigar sobre] ciertas enfermedades [como] en el caso de los medicamentos huérfanos o en el caso de los medicamentos para pocos pacientes, pues resulta un estímulo interesante para innovar, pero al mismo tiempo no se hacen otro tipo de políticas públicas en las que se destine mucho dinero del estado para esa misma innovación” (Colombia, Patient).  “Entonces eso sumado a los altos precios por un Estado, que en el caso colombiano, no intervino los precios, sino que al contrario desbordó absolutamente y permitió que se hiciera el abuso, pues esto sí originó un mayor gasto en un grupo de medicamentos, [...] una parte un poquito de biotecnológicos y algunos de estructura química conocida [...] Se llega a la judicialización por todas las variables que hemos tenido en cuenta y a eso se le suma que [el estado colombiano] ha sido [...] cómplice y ha vendido también su ética de ministros, de congresistas a la industria para permitir liberar los precios” (Colombia, Patient).  “Então, você pega o medicamento [oncológico] que saiu recentemente, que é monopólio, está sob patente, provavelmente esse procedimento que nós pagamos para o prestador não vai dar conta de financiar esse medicamento e acaba gerando ação judicial” (Brazil, Executive). | “Uma outra questão importante, que nós temos interesse por esse conjunto de medicamentos [do Componente Especializado da Assistência Farmacêutica - CEAF] é uma ação altamente estratégica, que é o seguinte, é o fortalecimento do complexo industrial da saúde [...] Esse componente [CEAF] é um componente que contribui muito para a política brasileira no campo do complexo industrial em saúde. Nós já estamos numa fase em que nós não estamos indo no mercado apenas para comprar medicamentos, nós estamos estimulando a produção nacional por meio de transferência tecnológica para produtos deste componente” (Brazil, Executive).  Lo importante [...] es que la Sentencia T-760 y el instrumento de supervisión del cumplimiento de esa sentencia, sí han permitido, digamos, acompañar toda la problemática desde el poder judicial, [incluyendo] la obligación de las autoridades gubernamentales de dar informes periódicos a la Corte, [...] hacen, digamos, seguramente por otras razones, que recientemente haya un cambio de la política pública sobre el control de los precios de los medicamentos, y la necesidad de que las empresas farmacéuticas no fijen, digamos, lo valores a su criterio, sino que el Estado está interesado por controlar ese tema (Colombia, Judicial). |
| Judiciary Power | “Entiendo que uno [el juez] a veces no cuenta con demasiados elementos, digamos, el juez entiende derecho, no entiende mucho, a veces, de medicina. Entonces, a veces, no tenemos los suficientes elementos, ya de por sí, como para resolver si corresponde o no el amparo, si es urgente o no. En mi experiencia lo que hago, es tratar de investigar por mi cuenta... me meto a internet y empiezo a averiguar, sobre si realmente es urgente la situación.... pero bueno, a veces, uno siempre opta por, ante la duda, de concederle a la persona este amparo” (Argentina, Judicial).  “Lo que piensa la justicia es [...] hay un médico que lo pide, esta persona lo necesita y hay alguien que se lo niega que es la empresa de medicina prepaga [o la obra social]. [...] Más allá de los argumentos que vos puedas tener como financiador, la justicia falla a favor de eso, del pedido y de la persona que lo necesita” (Argentina, manager).  “Eu quando judicializo, eu passo por cima de todas essas divisões [da organização do sistema de saúde]. Porque se elas não estão funcionando na prática, eu ignoro, entendeu? Porque na verdade, qual é nosso grande fundamento, que a Constituição Federal ela diz que a responsabilidade é da União do estado e do município, eles se organizarem internamente, eu acho ótimo, desde que esteja dando certo” (Brazil, Judicial).  “La Corte [Suprema] razona sobre la base que el derecho a la protección de la salud tiene que aportar los medios para protegerla [...] En Chile, yo diría que casi todos estos recursos de protección se ganan, yo he visto la situación en Uruguay y en Uruguay [...] prácticamente el 15% de los recursos de protección se ha ganado, el resto se pierden, el poder judicial está más alineado un poco con el estado o las aseguradores, en cambio acá [Chile] no, el poder judicial está muy alineado con los consumidores, con los usuarios, con los pacientes” (Chile, Lawyer). | “Porque esto perjudica [...] en lo judicial también, porque judicial nosotros, ya de por sí, estamos desbordados con la cantidad de causas judiciales que tenemos de toda índole ¿no? tanto civil, como penal. Entonces todos estos casos que empiezan a llegar de esta manera por supuesto suman, a lo que ya el poder judicial tiene” (Argentina, professional).  “Yo creo que un juzgado que no debería estar recibiendo ese número [de acciones judiciales], es un trabajo adicional en horas/hombre sí? Y eso implica que debe tener más personas, porque [el juzgado] debe responder primero en 10 días, porque [la acción judicial] es [sobre] salud y segundo las [acciones judiciales] que colocan medida pre-cautelar [el plazo para responder] es en 24 horas, entonces obviamente eso implica un recargo más administrativo de la gente de la rama judicial, en lo cual creo que nadie lo proyectó... [Además] nadie tampoco previó que se le está perdiendo el respeto a la tutela [...] evolucionamos en que ya nadie cumple la tutela, y ya la Corte [Constitucional] se tiene que pronunciar frente a una tutela de desacato” (Colombia, patient).  “A via judicial, embora fosse muito utilizada, não era uma garantia de acesso [a medicamentos], por quê, porque o estado não conseguia dar conta” (Brazil, manager). |
| Judiciary Power (cont.) | [En Colombia]... “ha habido un tema que uno podría llamar de movilización jurídica, o sea, el reconocimiento del derecho [a la salud] en la Constitución [...] y los jueces [tienen] una idea de conceder el derecho [a la salud] con la idea de que los derechos sociales también son protegibles por vía judicial”. (Colombia, NGO) |  |

**Table S3. Examples of causes and consequences related to the Local level**

| **Category** | **Causes** | **Consequences** |
| --- | --- | --- |
| Citizen rights holder | “La gente no se esmera en conocer a qué tiene derecho” (Colombia, Executive).  “Esa conciencia del derecho y [de] que el mecanismo de la tutela funciona rápido, eficaz, para obtener concretamente el medicamentos, entonces genera como esa idea de ‘yo tengo ese derecho, entonces yo lo puedo reclamar, si [las EPS] me lo niegan [el medicamento] yo lo reclamo’...” (Colombia, NGO).  “Hay en la sociedad una creencia, de que el poder judicial tiene las soluciones cuando el resto de los poderes [Ejecutivo y Legislativo] fallan” (Argentina, Judiciary). | “Considero totalmente positivo lo que ha sido el mejorar el acceso a medicamentos [...] y defender evidentemente el derecho de los pacientes que sí lo necesitan” (Colombia Professional).  “Para el paciente es importante, que ya entendió que tiene un derecho [...], que ya la mayoría de la gente conoce que hay un derecho y que hay un mecanismo para exigirlo” (Colombia, Patient).  “Un tema [...] que generan las prestaciones a través de amparo es que no es del todo equitativo, no sólo por lo que afecta el presupuesto para los restantes pacientes, sino también porque el acceso al amparo es para determinado grupo de población, que de algún modo se entera, tiene cierta llegada a distintos grupos de abogados, a distintas organizaciones, etc.” (Argentina, Executive). |
| Consumer of healthcare | “Con el avance evidentemente de la información que reciben los pacientes [...] los pacientes van cambiando [... de] Los que eran pacientes común denominador eran pacientes pasivos, podríamos decir: Doctor, ¿Qué será lo que tengo? ¿Qué es lo que me tengo que tomar o qué es lo que puedo tomar? A un paciente que dice tengo esto, tengo esta enfermedad, tengo que tomar este medicamentos, me hace la receta” (Argentina, Executive). | “Pero también hay otro problema, que es que no todas las personas tienen acceso a un abogado para hacer un recurso de protección, entonces el tema es absolutamente perjudicial para los pacientes” (Chile, lawyer).  “Outra questão que pode colocar dificuldade é a falta de segurança do usuário. Então [o juiz] ao tomar a decisão de atender a uma determinada ação, por exemplo, de um medicamento que já foi incorporado no SUS [pero para indicações não consideradas no CEAF], isso pode colocar em xeque a própria segurança do usuário” (Brazil, Executive). |
