# Supplementary material for: Judicialization of access to medicines in four Latin American countries: a comparative qualitative analysis
Source: Int J Equity Health. 2019 Jun 3;18:68. doi: 10.1186/s12939-019-0960-z (PMC6545681; doi:10.1186/s12939-019-0960-z)
Supplement: Supplementary file 3 — Quotes translated into English. (DOCX 27 kb) [file 12939_2019_960_MOESM3_ESM.docx]

**Table S1. Examples of quotes for the categories at International level**

| **Category** | **Causes** |
| --- | --- |
| **Right to health in the International Human Right treaties and essential medicines definition** | “… We all know that Big Pharma has been the most important lobbyist for pushing UN, WHO, everyone, to make the right to health a fundamental right in all countries, as it was clear [for pharmaceutical industry] that the people individually would not be able to buy and pay for the costs of their products, and the best thing about it was that the states have to pay for [the medicines]” (Colombia, Patient). |
| **The market and the Innovation model and intellectual property protection – TRIPS** | “I think that [judicialization] is closely related to the R&D model; and to how the pharmaceutical industry resolved the price issue very easily by means of what would be called third-party payer models. Thus, for them, it is no longer a problem that medicines may cost COP 600 million pesos or COP 700 million pesos patient/year, because in the end it is not the patient himself that pays, but the [health] system” (Colombia, professional). |

**Table S2. Examples of causes and consequences related to the National level categories**

| **Category** | **Causes** | **Consequences** |
| --- | --- | --- |
| Right to health in the Political Constitution | “There are many reasons [for judicialization]. First, fundamentally, the rules we have are very broad. Constitutional norms and treaties with constitutional status… which are very broad in terms of coverage, so virtually any patient that requires any benefits, because the rights are so broad, they [the rights] somehow allow them [the patients] to ask for [the benefits]” (Argentina, Executive).  "... [The new constitution] was much more open than the previous ones from the military regime […] it was a constitution that, for the first time in Brazil's history [in 1988], included some rights. Among them, the so-called Article 196 of the Constitution that, in a very broad and general way, provided health as a citizen's right and an obligation of the state, without defining it clearly "(Brazil, manager). | "But I would say that one of the most negative aspects [of judicialization] is [...] what I call [...] the pharmaceuticalization of the right to health, where it seems that everything is solved with medicines" (Colombia, University lecturer).  [In] “The *Tutela*... it is all about the person; the right [to health] is individual and not collective. So we're struggling for things of high cost, low impact on public health… I mean, there's a whole discussion of collective rights vs. the individual rights” (Colombia, manager). |
| Right to health in the Political Constitution (cont.) | “The Constitution of 1991 did two fundamental things in the country. It introduced the market in the provision of social services, public services in the country and, as a counterbalance, it guaranteed rights... it [the Constitution] made the citizens’ rights explicit, and created the *Tutela* mechanism to claim them in the case of… when it is considered that such rights have been violated” (Colombia, NGO). | “If [the patients] do not have money to hire a lawyer, they resort to the newspaper, to the media to denounce the situation [of lack of access to medicines]. They [the patients] do not accept to be discriminated because they are poor, because they do not have resources, since the state must resolve their problems” (Chile, lawyer). |
| Health system hardware | “The problem is… a more economic one, since the resources are scarce. So… the lack of protection… is a common denominator” (Argentina, Executive).  [One should consider] “which part of the problem [judicialization] is due to the EPS’s inefficiency, poor service provision or deficient management; and which part is due to a structural problem related to a human resources deficit, particularly regarding specialist and subspecialist” (Colombia, manager).  “Here [in Argentina], there is a National Administration since [president] Menen’s office, which is the ANMAT, National Administration of Medicines, Food and Technology, that determines, when certain standards are met, if a medicines can enter the market or not. But there is no one, no national organization, to tell if that [medicine] will be covered or not by the Social Security or the State, right? There is nothing. So, that depends on the free will, of any doctor [general or specialist] because doctors can prescribe what they want (Argentina, NGO). | “Listen, regarding [the impacts] on the *Obra Social*, sometimes the trouble is that [the judges] force you to provide a benefit; and for the *Obra Social*, obviously, it is not economically convenient because by being obliged to provide a certain part of the benefit, [the *Obra social*] ends up spending double or triple what you [the *Obra Social*] had to give... For the company, these [lawsuits] are prejudicial because the company spends twice more than what it was supposed to spend to cover the disease” (Argentina, Manager).  “From the State’s point of view, judicialization, in practice, calls forth a disorganization of the service. We have a lot of difficulties in handling the volume of lawsuits here in the state... We now have a concentration of lawsuits here; and this, from the point of view of the State and municipal health secretariats, represents an unmanageable volume; we cannot respond to the volume of lawsuits that we have here. The State’s structure is not scaled for that” (Brazil, manager). |
| Health system hardware (cont.) |  | “There is definitely a negative impact [of judicialization] on the financing of the [health] system, since the way people get access to the benefits not included in the benefit plan is messy, the system has the need to spend a lot of resources on therapeutic technologies, including medicines.... whose [costs] are very high and, as a consequence, the system has to allocate a great part of the resources” (Colombia, professional).  “There should be a state body that could supply these expensive medicines, with a budget defined as part of the fiscal budget, because ... you'll see, for a private entity, especially like us who does not seek profit.... in case we suffer one of these penalties [lawsuits] we would fall off the chair, because, as a consequence of these judgments, we would have to spend 20 million pesos (~USD 34.000) on a medicine, for one single person, and it is too much money for our budget, which must always tend towards a balance.” (Chile, Manager). |
| Health system software | “The second one [cause] is a perverse incentive that is generated by the creation of the EPS in Colombia. The perverse incentive, which is what kills them [EPS] and kills the way the system was designed in Colombia, is that ... [the nature] for profit of these companies implies that if they spend... if they supply fewer services [...] or spend less money, they can make more money... so there is a widespread opinion resulted from this contract model; it does not mean that EPS always does so, but whenever they deny or do not provide a service, people will believe that they are profit-oriented, that they want to make more money by denying the service” (Colombia, NGO).  “We noticed that there was certain resistance right at the entrance [of the health service unit], you see? ...in the sense that the public servant did not do what they had to do. So the public defender office [...] had a view that, in order to achieve prestige, it had to resort the Judiciary, so it filed lawsuits. So ... there was a favourable movement [at the defender office] but the servant did not want to do their job. Inadequate supply ... by the municipality, the state, somehow allows some favouritism to some pharmacy owned by someone kin to the health secretary or the mayor” (Brazil, Judiciary).  “[In the AUGE], for some pathologies the access to medicines is stratified into age ranges... In this case there are some things... for instance... [some people] within a certain age range are given the medicines and others are not ... So the access is not a standard for everyone, and there will be people who, for their age or [clinical] situation, will not have access to this program” (Chile, Patient organization). | [As a result of judicialization] “Public policy is exaggeratedly litigation-driven, and in the macro level of the health system, litigation is marginal in terms of access [...] less than 0.5% of the health actions are accessed [...] by judicial mechanisms” (Colombia, NGO).  “In the case of [medicines and services] not included in the POS (No POS), the contracting mechanisms of health services are *fee for service*, [...], nobody says anything, no one questions anything ... So, from the perspective of a healthcare provider or supplier [including the pharmaceutical industry] this is the most reasonable logic from the economic point of view, and this is covered by something called *Tutela* in Colombia”. (Colombia, manager).  “In the case of benefits which are included in the Mandatory Health Program, there is no doubt that they are in charge of the *Obra Social*, [...], and if something is taken to the Judiciary, [...] it is correct [...]. If the patient is not satisfied with what the *Obra Social* provides and [the medicine] is within the Compulsory Medical program, it is quite right that [the patient] resorts to the Judiciary, because a contract was somehow broken” (Argentina, manager).  “Well, a large number of individual lawsuits causes disorganization in their system [the agents responsible for the health care organization], so they start to do something. [...] They become so bothered that they actually start to act, to change the public policy, you see? So this is a really positive aspect” (Brazil, Judiciary). |
| Pharmaceutical marketing | "There is evidence about [the relationship between] pharmaceutical companies and doctors, who are somewhat influenced by the industry, [the doctors] also ended up going into that thing [litigation] as they realized that the Judiciary, let’s put it this way, looks favourably upon that sort of thing [litigation]" (Brazil, manager).  "But we must also see that there are vested interests behind them [patient organizations], which also led to judicialization, and yet important, right? In other words, the pharmaceutical companies are at times behind the patients and, a new medicine that has just come out... at the very next day, they [patients] are asking for it. You may think “But how can that be?” right? Of course, the pharmaceutical companies want to make up for the research costs, and want to put it [the medicine] on the market (Argentina, NGO).  The transnational [pharmaceutical] companies [...] will always push expensive drugs and will use all strategies do to so... from patient associations to the lawyers paid by such patient associations to demand the medicines. So, the transnational pharmaceutical industries are pushing the tutela (Colombia, NGO). | “Some interesting data that we had showed how some pharmaceutical companies took advantage by concentrating some very good and extremely profitable items and reimbursements, that is, it was clear that some companies were doing very well with the reimbursements” (Colombia, NGO). |
| National policies for science and technology development, intellectual property protection and medicines prices control | “When [innovation] is in private hands, we cannot know the value of that innovation [...] the interest in profit gets in the way of access, and somehow [...] these [developing] countries are compelled to meet the market rules, that is, to protect patents [...] which we believe is an encouragement for [carrying out research on] certain diseases [as] in the case of orphan medicines or in the case of the medicines for a few patients, because it ends up being an interesting incentive to innovate; but at the same time other public policies are not implemented so as to allocate a lot of money from the State for the same type of innovation.” (Colombia, Patient).  “So, all this combined with high prices by the State which, as in the case of Colombia, did not intervene in the prices but instead was absolutely open to abuse, as this indeed caused increased spending on a group of medicines [...] a part of few biotechnological medicines and some medicines of known chemical structure [...] as consequence of all the variables that we have taken into account and because the [Colombian] state has been [...] an accomplice and has also sold the ethics of ministers, congressmen, to the industry in order to allow free prices” (Colombia, Patient).  “So, take an [oncologic] medicine that was recently licensed as an example, which is under monopoly and under patent protection, this procedure, that we [ministry of health] pay to the [health care] provider, will probably not be enough to fund this medicine and it ends up generating a lawsuit” (Brazil, Executive). | “Another important issue that we have interest in this set of medicines [included in the Specialized Component of Pharmaceutical Assistance – CEAF] is a highly strategic action, which is the strengthening of the health industrial complex [...] This component [CEAF] contributes a lot to the Brazilian policy in the industrial complex of health. We are already at a stage where we are not going to the market just to buy medicines, but we are stimulating the national production through technological transfer for products of this component” (Brazil, Executive).  “The important thing [...] is that the Sentence T-760 and the instrument to monitor compliance of this sentence have indeed allowed to follow the whole issue from the Judiciary, [including] the obligation of government authorities to regularly submit reports to the Court [...] has recently brought about, shall we say, and certainly for other reasons, changes in the public policy on the control of medicine prices and the need that pharmaceutical companies do not fix the prices at their discretion, but the state is the one interested in controlling this issue” (Colombia, Judicial). |
| Judiciary Power | “I understand that one [the judge] sometimes does not have many elements, say, the judge understands about law, but sometimes not much about medicine. So, sometimes, we don’t have enough elements to decide whether the Amparo is appropriate or not if it is urgent or not. According to my experience, what I do is to try to investigate on my own ... I go to Internet and try to find out whether the situation is really urgent… but, well, you would always choose, when in doubt, for granting the Amparo to the person” (Argentina, Judicial).  “What the Judiciary thinks is that [...] there is a doctor who asks for it [the medicine], this person who needs it [the medicine], and there is someone who denies it, that is the prepaid medical company [or Obra Social]. [...] Beyond any arguments that you as a financier might have, the Judiciary will rule in favour of the request and the person in need” (Argentina, manage).  “When I recourse to the court, I step over all these divisions [of the health care organization] because if they, in practice, are not working, I ignore that, you see? For, in fact, what is our main foundation? The Federal Constitution says that such responsibility lies with the Union, the State and the municipality. And if they organize themselves internally, I think it's great, as long as it [this organization] turns out fine” (Brazil, Judicial).  “The [Supreme] Court reasons out on the basis that the right to health protection itself has to provide the means to protect it [health] [...] In Chile, I would say that most of these protection resources are won, and I have seen the situation in Uruguay, and in Uruguay [...] almost 15% of the protection resources have been won, and the remaining part has been lost, the Judiciary is a little more in line with the state or insurers, however not here [in Chile] where the Judiciary is closely aligned with consumers, users, patients” (Chile, Lawyer). | “Because this also affects [...] the Judiciary, as we, the Judiciary itself, are overwhelmed with the amount of lawsuits that we have of all kinds, right? Both civil and penal. Then all these cases that come this way will, of course, sum to those [lawsuits] that the Judiciary already has” (Argentina, professional).  "I believe that a court should not be taking this amount [of lawsuits]. This is additional work in hours/man, right? And that means that you need more people because it [the court] must respond first in 10 days, as it [the lawsuit] is [related to] health, and secondly the ones [lawsuits] that set a pre-cautionary measure [the deadline to respond] within 24 hours. This obviously implies more work charge for the Judiciary personnel, which I think no one had planned... [Moreover] no one has either foreseen that the Tutela is no longer respected [...] we evolved to the point that nobody complies with the Tutela, and the [Constitutional] Court has to pronounce against contempt for the Tutela"(Colombia, patient).  “The courts, although widely used, were not a guarantee of access [to medicines]. And why? Because the state was not able to account for that” (Brazil, manager). |
| Judiciary Power (cont.) | [In Colombia] “there has been an issue that could be called legal mobilization, that is, recognition of the right [to health] in the Constitution [...] and judges [have] an idea of granting the right [to health] with the idea that social rights are also protectable through the courts” (Colombia, NGO). |  |

**Table S3. Examples of causes and consequences related to the Local level**

| **Category** | **Causes** | **Consequences** |
| --- | --- | --- |
| Citizen rights holder | “People do not strive to know what rights they have.” (Colombia, Executive).  "This awareness of the right [to health] and [that] the protection of the Tutela works fast to effectively give access to the medicines, then it generates the idea of ‘I have that right, then I can claim it’, ‘If they [EPS] deny it [the medicine], I will claim it'..."(Colombia, NGO).  "There is a belief in society that the Judiciary has the solutions when the other branches [Executive and Legislative] fail" (Argentina, Judiciary). | “I consider the improvement in the access to medicines totally positive [...] and obviously is the defence of the right [to access to medicines] of patients that actually need them [the medicines]” (Colombia, Professional).  “It is important that patients understand they have a right [...], that most people know that there is a right and that there is a mechanism to demand it" (Colombia, Patient).  “One issue [...] about getting [access to] benefits by filing a Amparo is that it is not entirely fair, not only because it affects the budget for the remaining patients, but also because access to the Judiciary is just for a certain group of people who somehow gets to know about it and can get in contact with some lawyer groups or organizations, etc.” (Argentina, Executive). |
| Consumer of healthcare | “Obviously, with the advancement of information that patients receive, [...] they are changing [... from] a patient who was just a common denominator, who was a passive patient and would say “Doctor, what do I have? What [medicine] do I have to take or what I can take?” to a patient that says “I have this, I have this disease, I have to take this medicine, give me the prescription” (Argentina, Executive). | “But there is another problem, which is that not all people have access to a lawyer to file a protection resource, so the issue is absolutely detrimental to patients” (Chile, lawyer).  “Another issue that can create difficulty is the lack of safety for the user. So when [the judge] makes the decision to concede a particular benefit, for example, a medicine that has already been incorporated into the SUS [for indications not covered by the CEAF], this can put the user's own safety in check” (Brazil, Executive). |
